# Mitochondria‐Targeted Nanomotor: H_2_S‐Driven Cascade Therapy for Hepatocellular Carcinoma

**DOI:** 10.1002/adma.202513757

**Published:** 2025-12-01

**Authors:** Chengcheng Li, Xiaodong Ma, Shiji Fang, Biao Chen, Xinru Wang, Lingyun He, Xin Yang, Yuanqiang Li, Jessica M. Rosenholm, Zhongwei Zhao, Jiansong Ji, Hongbo Zhang

**Affiliations:** ^1^ Zhejiang Key Laboratory of Imaging and Interventional Medicine Zhejiang Engineering Research Center of Interventional Medicine Engineering and Biotechnology Department of Radiology Lishui Central Hospital The Fifth Affiliated Hospital of Wenzhou Medical University Lishui 323000 China; ^2^ Joint Centre of Translational Medicine, Wenzhou Key Laboratory of Interdiscipline and Translational Medicine the First Affiliated Hospital of Wenzhou Medical University Wenzhou 325000 China; ^3^ Pharmaceutical Sciences Laboratory, Faculty of Science and Engineering Åbo Akademi University Turku 20500 Finland; ^4^ Turku Bioscience Centre University of Turku and Åbo Akademi University Turku 20520 Finland

**Keywords:** cascade therapy, enzyme dynamic therapy, mitochondria‐targeting, photodynamic therapy, photothermal therapy

## Abstract

Despite advances in combination therapies for cancer treatment, most strategies rely on modular‐additive designs that lack dynamic molecular cues to achieve intrinsic synergy. Herein, a mitochondrial‐targeted nanoplatform is introduced that orchestrates photodynamic therapy (PDT), mild photothermal therapy (mPTT), and enzyme dynamic therapy (EDT) into a self‐amplifying cascade network through gasotransmitter (H_2_S)‐driven metabolic reprogramming. It is constructed from an Au_2_Pt core with a surface functionalized mesoporous silica shell loaded with photosensitizers, encapsulated within a tumor cell membrane (Au_2_Pt@4sMSN/PS‐TPP@CM). Upon GSH exposure, nanomotors produce H_2_S to boost diffusive motion, while TPP targeting directs this motility toward mitochondria, enabling efficient mitochondrial accumulation (internalization of >100 nm nanoparticles). Subsequently, mitochondrial targeted H_2_S releasing‐mediated suppression of oxidative phosphorylation amplifies PDT efficacy; HSP70 downregulation enables mPTT; and hyperactive glycolytic metabolism fuels EDT. Furthermore, these enhanced modalities also interconnect in a positive feedback loop: mPTT‐derived hyperthermia accelerates EDT‐catalyzed oxygen generation for PDT, while mitochondria‐localized PDT further inhibits HSP70 to boost mPTT. Ultimately, these interconnected molecular cues establish an H_2_S‐driven, self‐reinforcing therapeutic loop that enables effective eradication of hepatocellular carcinoma. Collectively, this study identifies mitochondria as the biological initiator and signal integrator for multimodal therapy, delivering a distinctive paradigm to overcome the limitations of conventional combination therapies.

## Graphical abstract

A mitochondrial‐targeted nanomotor integrates photodynamic therapy, mild photothermal therapy, and enzyme dynamic therapy into a self‐amplifying cascade through H_2_S‐driven metabolic reprogramming.

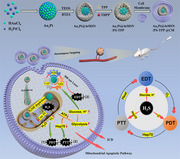

## 1 Introduction

Emerging cancer therapies, including photodynamic therapy (PDT), mild photothermal therapy (mPTT), enzyme dynamic therapy (EDT), and gas therapy (GT), offer novel advantages that circumvent the limitations of traditional treatments, such as drug resistance and systemic toxicity.^[^ ^1^ ^]^ However, the application of these monotherapies is still constrained by inherent limitations. For instance, PDT is spatially and temporally precise; it relies heavily on oxygen to generate the cytotoxic singlet oxygen, rendering it ineffective in hypoxic tumor microenvironments (TME).^[^ ^2^ ^]^ mPTT (41–43 °C) can upregulate heat shock protein 70 (HSP70), which mitigates thermal damage by refolding denatured proteins.^[^ ^3^ ^]^ EDT relies on catalytic reactions to generate therapeutic agents but faces challenges such as insufficient substrate and reduced enzymatic efficiency under pathological conditions.^[^ ^4^ ^]^ Similarly, GTs like hydrogen sulfide (H_2_S) can modulate redox homeostasis and apoptosis but suffer from issues such as short half‐life, poor targeting, and uncontrolled diffusion.^[^ ^5^ ^]^ Therefore, these monotherapies fail to meet the need for ideal anti‐tumor therapeutic efficacy.

To overcome the challenges in monotherapy, a range of enhanced therapy approaches have been developed, including HSP inhibitors‐enhanced PDT,^[^ ^6^ ^]^ oxygen‐carrying systems for hypoxia alleviation,^[^ ^7^ ^]^ and sustained H_2_S gas‐releasing nanoplatforms.^[^ ^8^ ^]^ However, these strategies rely on static delivery of exogenous modulators and lack dynamic responsiveness to endogenous biological cues, making them susceptible to compensatory adaptations triggered by the tumor microenvironment,^[^ ^9^, ^10^, ^11^ ^]^ ultimately reducing therapeutic effectiveness. Furthermore, most of the emerging combinational enhanced therapy platforms adopt a “modular additive” design where therapeutic components (e.g., photosensitizers, gas donors, or metallic catalysts) act separately without molecular crosstalk or synergy among each other,^[^ ^12^, ^13^ ^]^ failing to establish a multi‐modal therapeutic loop at the molecular level. Therefore, neither enhancement nor combination strategies adequately address the dynamic interplay between cancer cell defenses and microenvironmental stressors.

Interestingly, mitochondria, as the central hub of cellular metabolism and signal transduction,^[^ ^14^, ^15^ ^]^ integrate multiple functions including gasotransmitter signaling, energy production, redox homeostasis, and apoptotic regulation, thereby providing a robust biological foundation for centralized multimodal therapeutic synergy. Specifically, mitochondria serve as the primary targets for gasotransmitters such as H_2_S, which can suppress the maximal oxygen consumption rate (OCR) for 24–48 h by directly interacting with components of the electron transport chain.^[^ ^16^ ^]^ The energy required for the synthesis and chaperone activity of the heat shock protein family (HSP70/HSP90) is also predominantly supplied by ATP generated via oxidative phosphorylation.^[^ ^17^ ^]^ Furthermore, reactive oxygen species (ROS) generated during PDT primarily target the mitochondria to activate the intrinsic apoptotic pathway.^[^ ^18^ ^]^ Additionally, mitochondria‐mediated metabolic cycling offers dynamically replenishable substrates, making it ideal for enhancing nanoenzyme‐driven EDT.^[^ ^19^, ^20^ ^]^ These features collectively establish mitochondria as a central biological nexus for integrating GT, mPTT, PDT, and EDT, enabling a dynamically synergistic and logically programmed multimodal therapeutic strategy.

**Scheme 1:**
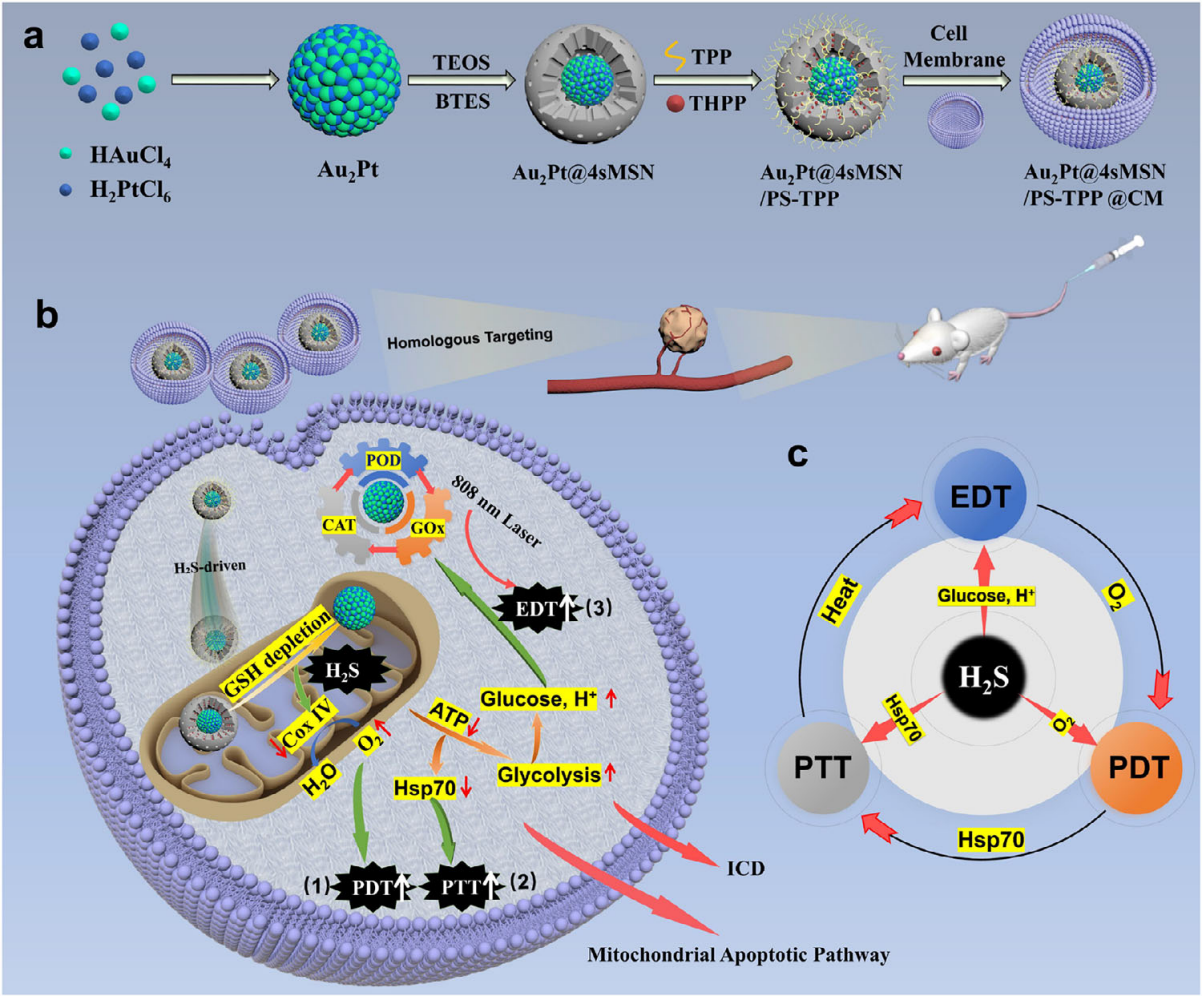
Scheme of the preparation and anti‐tumor mechanism of Au_2_Pt@4sMSN/PS‐TPP@CM. a) Schematic illustration of the preparation process for the mitochondria‐targeting nanoplatform Au_2_Pt@4sMSN/PS‐TPP@CM. b) Mechanistic elucidation of molecular interplay‐driven multimodal therapeutics mediated by Au_2_Pt@4sMSN/PS‐TPP@CM. c) Mechanistic schematic of the H_2_S‐driven self‐reinforcing therapeutic amplification loop.

Static delivery of exogenous modulators fails to address the complexity and adaptive compensation mechanisms of TME, and simple additive combinations even elicit antagonistic effects. Efficient therapeutic synergy demands a dynamic, context‐responsive system capable of sensing and adapting to the TME's evolving biochemical cues. Building upon this concept, we developed a mitochondria‐targeted nanoplatform, Au_2_Pt@4sMSN/PS‐TPP@CM (**Scheme** **1**a), which orchestrates a self‐reinforcing cascade of GT, EDT, PDT, and mPTT in response to spatiotemporally varying molecular signals within the TME. This system incorporates: 1) an Au_2_Pt alloy nanomaterial core exhibiting triple enzymatic activity (peroxidase (POD), catalase (CAT), and glucose oxidase (GOx)); 2) a tetrasulfide bond‐bridged mesoporous silica shell (4sMSN) for GSH‐responsive H_2_S release; 3) tetrakis(4‐hydroxyphenyl) porphyrin (THPP) photosensitizers (PS) loaded within the mesopores; 4) surface modification with triphenylphosphine (TPP) for mitochondria targeting; and 5) a tumor cell membrane (CM) coating for homologous targeting.

Upon internalization by tumor cells (Scheme 1b), the nanoparticles are enriched at the mitochondrial periphery through TPP‐mediated targeting. Elevated GSH can break the tetrasulfide bonds in the 4sMSN shell to trigger localized H_2_S generation. Subsequently, the nanoparticles are propelled into the mitochondria via H_2_S‐enhanced diffusive motion. Simultaneously, H_2_S releases further disrupting mitochondrial respiration and suppressing oxidative phosphorylation (OXPHOS) of mitochondria. It will decrease oxygen consumption, thereby alleviating tumor hypoxia to enhance PDT, and then reduce ATP‐dependent HSP70 synthesis to sensitize cancer cells to mPTT. Subsequently, tumors driven by metabolic heterogeneity and compensatory regulation shift from OXPHOS to a hyperactive glycolysis phenotype as an adaptive resistance mechanism against H_2_S‐based GT. This metabolic reprogramming paradoxically creates favorable conditions for Au_2_Pt nanozymes with EDT (triple‐enzyme activities: POD, CAT, GOx), enabling synergistic starvation therapy and chemodynamic therapy (CDT). Moreover, the enhanced PDT, mPTT, and EDT modalities cooperatively establish a positive‐feedback therapeutic loop (Scheme 1c) to damage mitochondrial integrity and function, thereby activating the intrinsic apoptotic pathway along with immunogenic cell death (ICD) for efficient tumor eradication.

Collectively, this nanoplatform transcends conventional additive strategies, enabling a truly synergistic molecular interplay that reprograms the tumor microenvironment from a passive barrier into an active facilitator of therapeutic efficacy. It represents a technical and conceptual advance in precision nanomedicine and cascade therapy, delivering a novel paradigm for dynamically synergistic and logically programmed multimodal therapeutic strategy against hepatocellular carcinoma.

## 2 Results

### 2.1 Synthesis and Characterization of Au_2_Pt@4sMSN/PS‐TPP@CM

**Figure 1:**
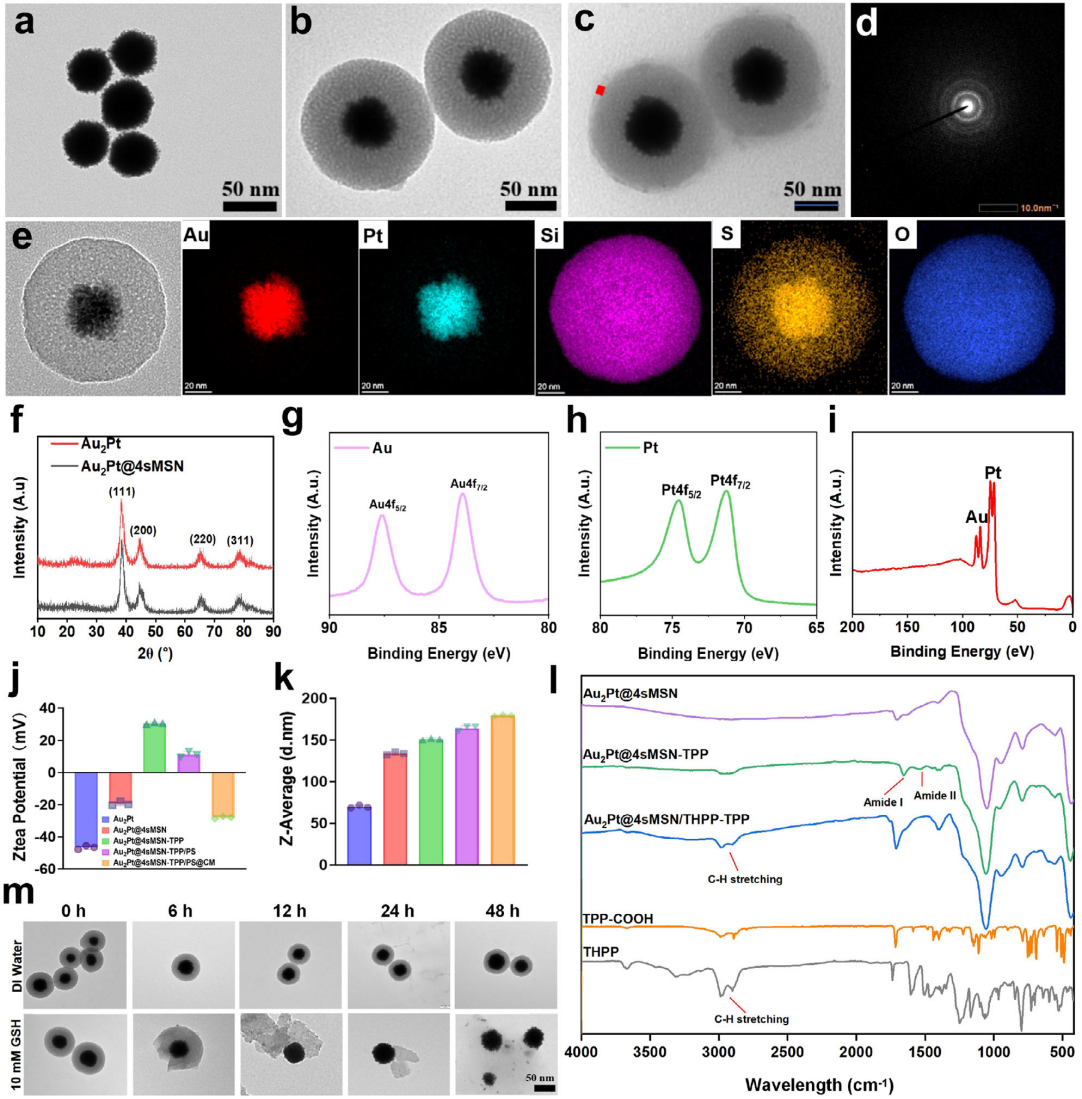
Synthesis and characterization of Au_2_Pt@4sMSN/PS‐TPP@CM. a) The TEM image of Au_2_Pt, b) Au_2_Pt@4sMSN, c) Au_2_Pt@4sMSN/PS‐TPP@CM (the rectangle: the thickness is 5 nm). d) Selected area electron diffraction (SAED) of Au_2_Pt. e) Mapping images of Au_2_Pt@4sMSN. f) XRD pattern of Au_2_Pt and Au_2_Pt@4sMSN. g–i) XPS spectra of Au_2_Pt. j) The zeta potential of various nanoparticles. k) The hydrodynamic diameter of various nanoparticles. l) FTIR spectrum of various nanoparticles. m) GSH‐responsive degradation of Au_2_Pt@4sMSN/PS‐TPP@CM.

The synthetic process of Au_2_Pt@4sMSN/PS‐TPP@CM is shown in Scheme 1a. First, the Au_2_Pt nanozyme was synthesized via a one‐pot method according to a previous report.^[^ ^21^ ^]^ PtCl_6_^2−^ and AuCl_4−_ were co‐reduced using L‐ascorbic acid, with L‐proline introduced as a chelating agent to slow the reduction kinetics and promote the formation of well‐dispersed Au_2_Pt nanozymes. **Figure** **1**a shows the TEM image of an Au_2_Pt nanoparticle, which has a rough‐surfaced spherical structure with a diameter of ≈50 nm. The hydrodynamic diameter was ≈70 nm (Figure 1k), measured by dynamic light scattering (DLS). As the X‐ray diffraction (XRD) pattern shown in the Figure 1f, Au_2_Pt nanozymes displayed characteristic peaks at 38.52°, 44.57°, 65.40°, and 77.89°, corresponding to the (111), (200), (220), and (311) planes of the face‐centered cubic phase of Au_2_Pt, respectively, providing strong evidence for the crystalline structure of Au_2_Pt. X‐ray photoelectron spectroscopy (XPS) analysis further confirmed the presence of both Au⁰ and Pt⁰ in the Au_2_Pt alloy (Figure 1g–i), validating the alloy composition of the nanoparticles. Additionally, the result of selected area electron diffraction (Figure 1d) demonstrated the polycrystalline structure of Au_2_Pt alloy with the diffraction rings, further confirming the successful fabrication of Au_2_Pt nanozyme.

Subsequently, the Au_2_Pt nanospheres were uniformly coated with tetrasulfide bond‐bridged MSNs (4sMSN) according to a previously established method with minor modifications.^[^ ^22^ ^]^ The TEM image (Figure 1b) of Au_2_Pt@4sMSN shows that the shell thickness is ≈40 nm and pore sizes range from 2.5 to 4 nm, which enables continuous GSH consumption for H_2_S release and supports a higher drug‐loading capacity. The hydrodynamic diameter changed to ≈133 from 70 nm for the core only. Furthermore, the elemental mapping results (Figure 1e) indicated that Au and Pt were localized in the core region, while Si, S, and O were distributed throughout the whole nanoparticle, confirming the encapsulation of 4sMSN onto the Au_2_Pt core. Although the tetrasulfide‐bridged silica shell remains morphologically intact, the sulfur signal detected in the Au_2_Pt core in the EDS mapping is most likely an artefact originating from background and peak overlap between S and the M‐lines of Au/Pt. Additionally, the EDS spectrum (Figures S1 and S2, Supporting Information) further reveals the elemental composition and spatial distribution, confirming that Au_2_Pt@4sMSN was successfully synthesized. As shown in Figure 1f, the crystal structure of Au_2_Pt@4sMSN remained unchanged compared to its precursor Au_2_Pt, indicating that the shell coating also does not compromise the integrity of the nanozyme. XPS spectra (Figure S3, Supporting Information) and atomic ratio (Figure S4, Supporting Information) of Au_2_Pt@4sMSN further reveal the presence of Au⁰ and Pt⁰, reinforcing this conclusion.

To impart mitochondrial targeting functionality to Au_2_Pt@4sMSN, TPP, a classic mitochondria‐targeting ligand, was covalently conjugated onto the 4sMSN surface via an amination reaction. The FITC spectrum (Figure 1l) displays pronounced amide I (≈1650 cm^−1^) and amide II (≈1525 cm^−1^) absorption peaks, confirming the formation of amide bonds and thereby verifying the successful conjugation. Furthermore, the observed increase in hydrodynamic diameter from 134.2 to 153.9 nm, along with a shift in zeta potential from −19.3 to +30.6 mV (Figure 1k,j), provides additional evidence supporting the successful surface modification.

Tetrakis(4‐hydroxyphenyl) porphyrin (THPP), as a photosensitive agent (PS), was subsequently loaded into the mesoporous architecture of the 4sMSN shell. The reduced zeta potential (from +30.6 to +11.2 mV), along with characteristic spectral changes in the FTIR spectrum (C–H stretching), confirmed successful PS loading. Finally, to further achieve homologous tumor targeting, the extracted Hepa 1‐6 cell membrane was self‐assembled onto the Au_2_Pt@4sMSN/PS‐TPP surface using a previously reported method.^[^ ^23^ ^]^ TEM imaging (Figure 1c) revealed a membrane thickness of ≈5 nm, and the zeta potential decreased to ≈‐28 mV after the CM coating.

The structural stability and GSH‐responsive behavior of Au_2_Pt@4sMSN/PS‐TPP were evaluated by incubating the nanoparticles in PBS solution or PBS solution containing 10 mM GSH at pH 6.5 for 48 h. TEM results (Figure 1m) demonstrated that the nanostructure remained stable under PBS solution, while the 4sMSN shell gradually degraded in response to GSH exposure. Taken together, these results collectively confirm the successful synthesis of Au_2_Pt@4sMSN/PS‐TPP and its GSH‐responsive degradability.

### 2.2 Characterization of GT Mediated by Au_2_Pt@4sMSN/PS‐TPP@CM

**Figure 2:**
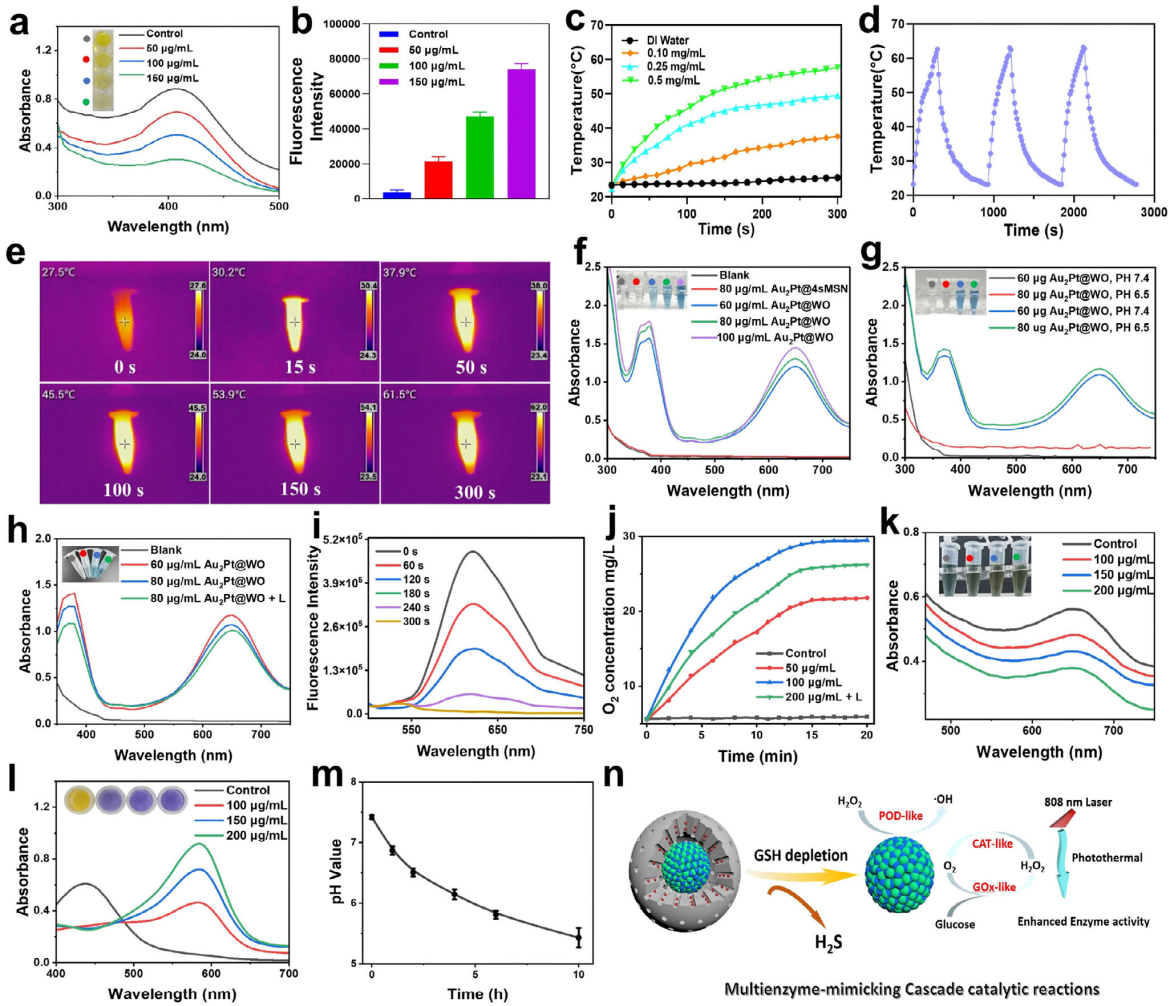
Characterization of GT, mPTT, and EDT mediated by Au_2_Pt@4sMSN/PS‐TPP@CM in vitro. a) GSH depletion ability of Au_2_Pt@4sMSN/PS‐TPP@CM. b) Fluorescence intensity detection of H_2_S generation. c) Temperature change curve of Au_2_Pt@4sMSN/PS‐TPP@CM at various concentrations. d) Temperature variation curve during the three turn‐on/off cycles of Au_2_Pt@4sMSN/PS‐TPP@CM. e) Infrared thermography images of Au_2_Pt@4sMSN/PS‐TPP@CM under 808 nm laser irradiation. f) POD‐like enzyme activity of Au_2_Pt@4sMSN and Au_2_Pt@WO. g) POD‐like enzyme activity of Au_2_Pt@WO under different pH values. h) POD‐like enzyme activity of Au_2_Pt@WO under 808 nm laser irradiation. i) Fluorescence spectrum of oxygen generation of Au_2_Pt@WO. j) Oxygen generation curve of Au_2_Pt@WO at different concentrations or under 808 nm laser irradiation. k) Glucose consumption ability of Au_2_Pt@WO. l) H_2_O_2_ generation after the incubation of Au_2_Pt@WO with glucose solution. m) pH change curve following the incubation of Au_2_Pt@WO with glucose solution. n) Schematic of three‐enzyme cascade activity of Au_2_Pt@WO.

The tetrasulfide bond‐bridged 4sMSN shell of Au_2_Pt@4sMSN/PS‐TPP@CM is selectively cleaved by GSH in the TME, generating GSSH and H_2_S. This reaction not only depletes intracellular GSH but also enables sustained H_2_S release, thereby activating GT. To evaluate GSH‐triggered H_2_S generation in vitro, Au_2_Pt@4sMSN/PS‐TPP@CM was incubated with 10 mM GSH for 4 h, and the residual GSH content was then measured. As shown in **Figure** **2**a, higher concentrations of Au_2_Pt@4sMSN/PS‐TPP@CM resulted in greater GSH consumption. The depletion of GSH suggests the generation of H_2_S, which is supported by optical images in Figure S5 (Supporting Information), showing an increase in gas bubbles with increasing concentration.

WSP5 is a fluorescein‐based probe that reacts selectively with H_2_S to generate a fluorescent product, which emits green fluorescence at 525 nm upon excitation, thus enabling sensitive detection of H_2_S levels. As shown in Figure 2b, the fluorescence intensity at 525 nm of the reaction solution increased proportionally with Au_2_Pt@4sMSN/PS‐TPP@CM concentration, confirming dose‐dependent H_2_S release. Additionally, the time‐dependent degradation behavior was also shown in Figure 1m. TEM images revealed that the 4sMSN shell degrades progressively over time and completely disappears after 48 h. Therefore, these results demonstrate the excellent GSH‐triggered H_2_S release properties of Au_2_Pt@4sMSN/PS‐TPP@CM, enabling H_2_S‐mediated gas therapy against hepatocellular carcinoma.

### 2.3 Characterization of mPTT Mediated by Au_2_Pt@4sMSN/PS‐TPP@CM

Au exhibits strong photothermal properties upon 808 nm laser irradiation and has been widely applied in mPTT treatment. To assess the photothermal performance of Au_2_Pt@4sMSN/PS‐TPP@CM, nanoparticles were dispersed in water at different concentrations (0, 100, 250, 500 µg mL^−1^) and irradiated with an 808 nm laser (1 W cm^−^^2^) for 5 min. The temperature changes were recorded using an infrared thermal camera. As depicted in Figure 2c, Au_2_Pt@4sMSN/PS‐TPP@CM exhibited a concentration‐dependent temperature elevation, with a maximum rise of 34.3 °C at 0.5 mg mL^−1^, whereas pure water showed only a minor increase of 2.2 °C, confirming the superior photothermal conversion capability of Au_2_Pt@4sMSN/PS‐TPP@CM. Furthermore, the temperature rise exhibited a clear dependence on laser power, as tested at 500 µg mL^−1^ concentration (Figure S6, Supporting Information). Notably, the photothermal performance of Au_2_Pt@4sMSN/PS‐TPP@CM remained stable across three consecutive laser on/off cycles, indicating its excellent photothermal stability (Figure 2d). Additionally, the thermal imaging capabilities of Au_2_Pt@4sMSN/PS‐TPP@CM under 808 nm laser irradiation were further confirmed (Figure 2e), with the maximum temperature reaching 61.3 °C at 0.5 mg mL^−1^. These results demonstrate that Au_2_Pt@4sMSN/PS‐TPP@CM is a highly promising candidate for mPTT in the NIR bio‐window due to its stable and controllable photothermal performance.

### 2.4 Characterization of EDT Mediated by Au_2_Pt@4sMSN/PS‐TPP@CM

The 4sMSN coating can effectively shield the catalytic site of the nanozyme core, thereby inhibiting Au_2_Pt‐mediated EDT activity. This strategy can not only improve the nanoplatform's biocompatibility but also impart spatiotemporal responsiveness to the EDT process, facilitating its dynamic adaptation to the tumor's metabolic shift toward a hyperactivated glycolytic phenotype. To mimic the spatiotemporal activation, Au_2_Pt@4sMSN/PS‐TPP@CM nanoparticles were pre‐incubated with 10 mM GSH for 24 h. The resulting particles with the shell removed (designated as Au_2_Pt@WO) were collected for subsequent evaluation of multienzyme‐mimicking activities (POD, CAT, and GOx‐like functions).

To assess the POD‐like activity of Au_2_Pt@WO, 3, 3′, 5, 5′‐Tetramethylbenzidine (TMB) was used as a peroxidase substrate, which is oxidized into oxTMB (blue color) with an absorption peak at 652 nm under peroxidase catalysis.^[^ ^24^ ^]^ As shown in Figure 2f, the amount of generated ^•^OH increased with the concentration of Au_2_Pt@WO, resulting in a deeper blue color. In contrast, the color of the Au_2_Pt@4sMSN group was similar to that of the blank, without POD enzyme activity, indicating that the 4sMSN shell effectively isolated the catalytically active sites of Au_2_Pt. This property of Au_2_Pt@4sMSN enabled the targeted chemodynamic therapy (CDT) with reduced toxicity to healthy cells, as the tetrasulfide bond‐bridged shell exhibits rapid degradation specifically in tumor cells with high GSH levels. Then, the effect of pH on the POD‐like activity of Au_2_Pt@WO was further investigated. According to Figure 2g, the enzyme activity was enhanced with decreasing pH, while there was no POD enzyme activity at pH 7.4. This suggests that Au_2_Pt exhibited improved POD‐like enzyme activity in more acidic environments, such as in the tumor microenvironment, but maintains good biocompatibility at the normal tissue microenvironment at pH 7.4. More interestingly, the POD activity can also be improved by photothermal treatment (Figure 2h). An increase in POD‐like enzymatic activity was detected under 808 nm laser irradiation.^[^ ^25^ ^]^ Notably, we further compared the POD‐like activities of Au_2_Pt@WO and Au_2_Pt, as depicted in Figure S7 (Supporting Information). A discernible reduction in catalytic activity was observed for Au_2_Pt@WO, presumably attributed to partial shielding of the active surface sites by thiol bonds. Collectively, these results demonstrate that Au_2_Pt@WO exhibits excellent POD‐like catalytic activity, which can be further enhanced under acidic conditions or upon 808 nm NIR laser exposure.

The CAT‐like activity of Au_2_Pt@WO was assessed by monitoring oxygen release. As illustrated in Figure 2i, the oxygen concentration over time was verified using a luminescent oxygen sensor probe Ru(dpp)_3_Cl_2_. The progressive decrease in the fluorescence peak at 613 nm indicated continuous oxygen generation. Figure 2j presents the oxygen production capacity of Au_2_Pt@WO in the presence of H_2_O_2_, revealing that higher concentrations of Au_2_Pt@WO generated more oxygen within the same reaction conditions. Again, the 808 nm laser irradiation had significantly accelerated the reaction rate and increased the yield of oxygen. This result was further corroborated by optical images (Figure S8, Supporting Information), where a greater number of bubbles were formed in solutions with higher sample concentrations or upon 808 nm NIR laser irradiation.

Finally, the GOx‐like activity of Au_2_Pt@WO was examined by assessing its ability to catalyze glucose oxidation, producing gluconic acid and hydrogen peroxide. As demonstrated in Figure 2k, higher concentrations of the Au_2_Pt@WO led to increased glucose depletion, along with a corresponding rise in H_2_O_2_ production (Figure 2l) and a decline in pH value (Figure 2m). Notably, under 808 nm laser irradiation, glucose consumption was further enhanced, as demonstrated in Figure S9 (Supporting Information). These findings confirm that Au_2_Pt@WO efficiently catalyzes glucose oxidation, and laser irradiation further enhances its GOx‐mimetic enzymatic activity.

Taken together, Au_2_Pt@WO exhibits highly efficient POD‐like, CAT‐like, and GOx‐like activities, demonstrating its multifunctional enzymatic properties (Figure 2n). Moreover, previous study have validated that the interdependent substrate–product conversions can further enhance the catalytic efficiency of Au_2_Pt, endowing the system with a “three‐in‐one” functionality to initiate a cascade catalytic reaction^[^ ^26^ ^]^ Specifically, the GOx‐like enzyme activity catalyzes the oxidation of glucose to generate H_2_O_2_ and H⁺, where the produced H_2_O_2_ serves as a substrate for CAT, and the accompanying pH reduction enhances POD activity. Meanwhile, CAT‐mediated oxygen generation provides a continuous supply of substrate for GOx, thereby establishing a self‐sustained catalytic cascade. This self‐cascade catalytic performance of Au_2_Pt@WO renders it a highly promising candidate for EDT in the treatment of hepatocellular carcinoma.

### 2.5 Biocompatibility of Au_2_Pt@4sMSN/PS‐TPP@CM

**Figure 3:**
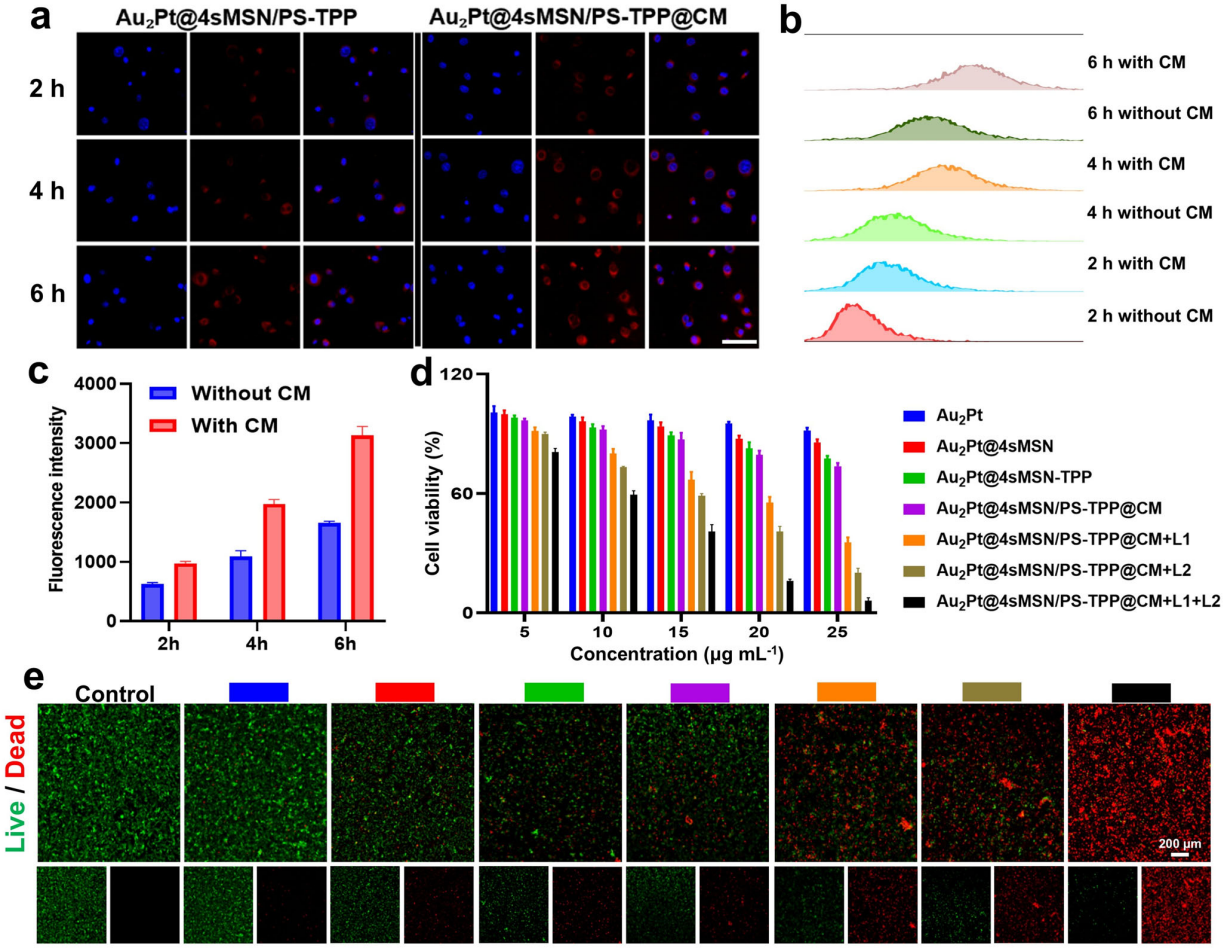
The biocompatibility of Au_2_Pt@4sMSN/PS‐TPP@CM. a) Fluorescence images of Hepa1‐6 cells incubated with Au_2_Pt@4sMSN/PS‐TPP and Au_2_Pt@4sMSN/PS‐TPP@CM for 2, 4, and 6. The scale bar is 100 µm. b) Flow cytometry analysis of cellular uptake. c) Flow cytometric quantification of nanoparticle internalization. d) CCK‐8 assay of different nanoparticles with various treatments. e) Calcein‐AM and PI fluorescence images of Hepa1‐6 cells with various treatments.

The above results have demonstrated the in vitro performance of GT, EDT, PDT, and mPTT mediated by Au_2_Pt@4sMSN/PS‐TPP@CM. Subsequently, the cell membrane‐mediated homologous targeting of Au_2_Pt@4sMSN/PS‐TPP@CM was analyzed using confocal laser scanning microscopy (CLSM). After incubation with Hepa1‐6 cells (**Figure** **3**a) for various time points (2, 4, and 6 h), a time‐dependent increase in intracellular fluorescence intensity was observed, suggesting efficient cellular uptake. Notably, the uptake efficiency was significantly higher in the Au_2_Pt@4sMSN/PS‐TPP@CM group compared to the group without cell membrane (Au_2_Pt@4sMSN/PS‐TPP) modification (Figure S10, Supporting Information). This was further confirmed by flow cytometry analysis (Figure 3b), which showed a higher fluorescence intensity (Figure 3c) in the presence of the CM coating group, indicating enhanced internalization. This improvement is likely due to the adhesion molecules retained on the cell membrane surface, which promote the internalization process.^[^ ^27^ ^]^

We further evaluated the biocompatibility of the nanoparticles in both Hepa1‐6 cells and NIH/3T3 cells using the CCK‐8 assay. As shown in Figure 3d, at a concentration of 20 µg mL^−1^, Au_2_Pt@4sMSN/PS‐TPP@CM exhibited selective cytotoxicity toward Hepa1‐6 cells, while maintaining high biocompatibility with NIH/3T3 cells (Figure S11, Supporting Information). It was attributed to the rapid GSH‐driven degradation of the nanoparticle shell in Hepa1‐6 cells, which generated sufficient H_2_S and activated the enzyme‐like activity of Au_2_Pt core to initiate CDT, thereby increasing oxidative stress and inducing apoptosis. Conversely, when co‐incubating with NIH/3T3 (normal) cells, which do not exhibit a micro‐acidic environment or high GSH concentrations, the nanoparticle showed minimal degradation of the shell, resulting in higher biocompatibility. Additionally, Au_2_Pt only exhibits POD‐like activity under acidic pH and sufficient hydrogen peroxide conditions,^[^ ^26^ ^]^ further contributing to the biosafety of the nanoplatform in normal tissues.

Furthermore, we applied both 808 nm laser and 660 nm laser irradiation to the nanoparticle‐treated cells. As compared to 808 nm laser (L1) irradiation, 660 nm (L2) laser irradiation treatment induced a greater reduction in cell viability, likely due to the limited therapeutic efficacy of mPTT. The combination of 808 nm and 660 nm laser irradiation resulted in the greatest therapeutic effect, leading to a reduction in cell viability to 16.07% with 20 µg mL^−1^ of Au_2_Pt@4sMSN/PS‐TPP@CM. Calcein‐AM/PI staining (Figures 3e; S12, Supporting Information) further confirmed that cell toxicity was significantly enhanced under 808 and 660 nm laser irradiation at Au_2_Pt@4sMSN/PS‐TPP@CM concentration of 20 µg mL^−1^. The cell viability of the Au_2_Pt@4sMSN/PS‐TPP@CM + L1 + L2 group decreased significantly to 13.47%, compared to 74.71% in the non‐irradiated group, indicating the significant synergistic effects of EDT, PDT, mPTT, and GT. Based on these toxicity data, a concentration of 20 µg mL^−1^ was selected for subsequent in vitro anti‐tumor mechanism validation.

### 2.6 H_2_S‐Driven Mitochondrial Targeting Ability of Au_2_Pt@4sMSN/PS‐TPP@CM

**Figure 4:**
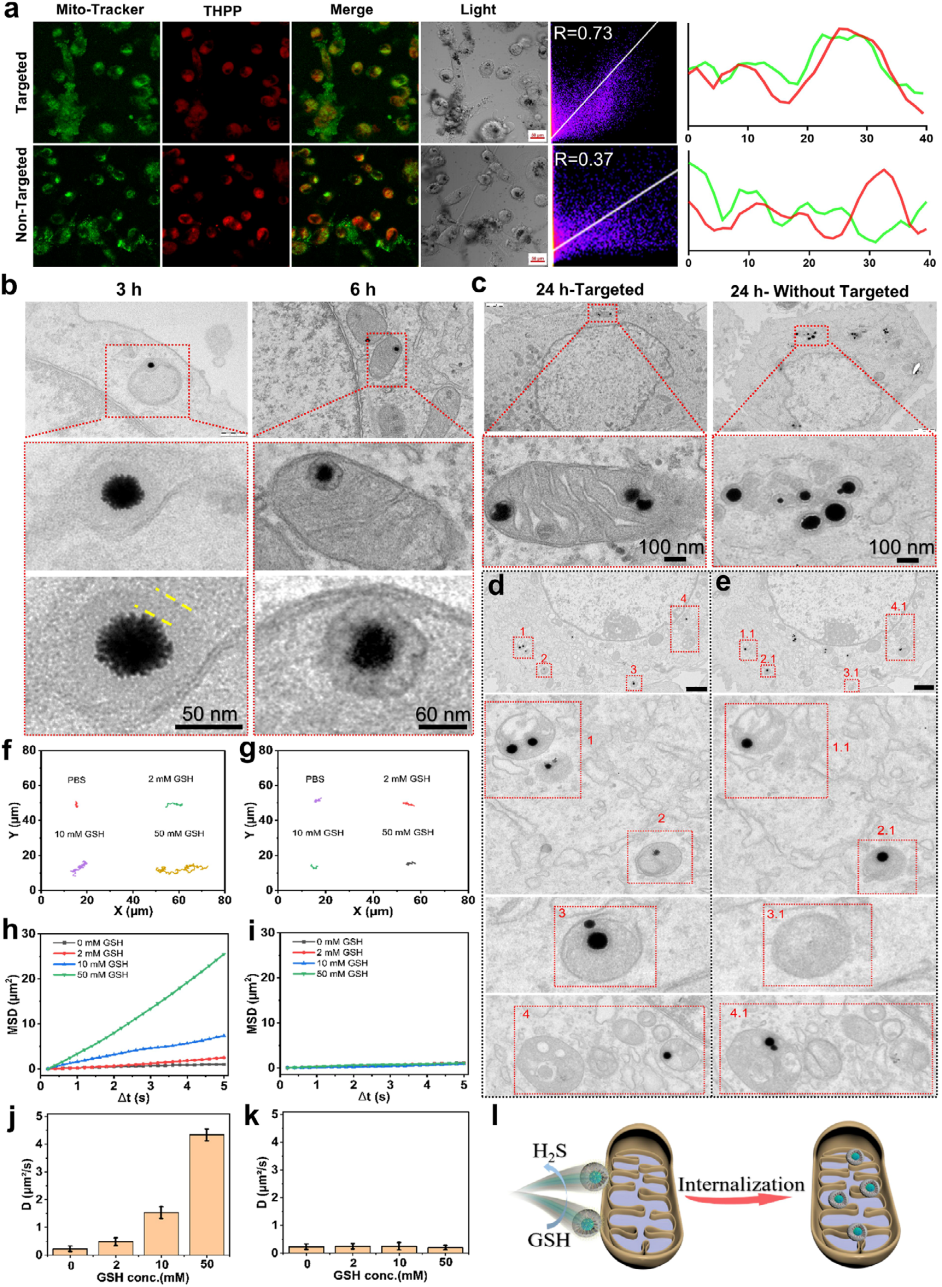
The H_2_S‐driven mitochondrial targeting ability of Au_2_Pt@4sMSN/PS‐TPP@CM. a) Mitochondrial‐targeting fluorescence images and colocalization analysis of Au_2_Pt@4sMSN/PS‐TPP@CM and Au_2_Pt@4sMSN/PS@CM. b) Bio‐TEM images of Au_2_Pt@4sMSN/PS‐TPP@CM nanoparticles internalized by Hepa 1‐6 cells (4sMSN shell demarcated by the yellow dashed line). c) Bio‐TEM images of Au_2_Pt@4sMSN/PS‐TPP@CM and Au_2_Pt@4sMSN/PS@CM nanoparticles after 24 h of incubation. d,e) Bio‐TEM images of serial ultrathin sections (70 nm) from the targeted group. The red dashed rectangles represent the same location from sequential sections. The bar is 1µm. f) The diffusion trajectories of Au_2_Pt@4sMSN and g) Au_2_Pt@MSN in the presence of GSH. h) The mean square displacement results indicate the dynamic behaviors of Au_2_Pt@4sMSN and i) Au_2_Pt@MSN under GSH exposure. j) The diffusion coefficients of Au_2_Pt@4sMSN and k) Au_2_Pt@MSN. l) Schematic illustration of H_2_S‐driven mitochondrial internalization of nanoparticles.

Subsequently, to further assess the mitochondrial localization efficiency of Au_2_Pt@4sMSN/PS‐TPP@CM, CLSM imaging (**Figure** **4**a) was conducted using MitoTracker Green as a mitochondrial marker. The results showed a strong colocalization between Au_2_Pt@4sMSN/PS‐TPP@CM and MitoTracker Green fluorescence, with a Pearson's correlation coefficient (R) of 0.73, indicating a high affinity for mitochondria. In contrast, the group without TPP modification showed a lower Pearson's correlation coefficient (R = 0.37). The line profile analysis further supported the role of TPP in enhancing mitochondrial targeting.

To directly visualize the mitochondrial targeting capability of the nanoplatform, Au_2_Pt@4sMSN/PS‐TPP@CM nanoparticles were co‐incubated with Hepa1‐6 cells for 3 and 6 h and subsequently subjected to biological transmission electron microscopy (Bio‐TEM) imaging after embedding in ultra‐thin slices. As shown in Figure 4b, the nanoparticles appeared to be undergoing mitochondrial internalization, as evidenced by the fusion between the mitochondrial bilayer and the nanoparticle shell. Moreover, nanoparticles with partially degraded shells were observed within the mitochondria at 6 h. Figure 4c shows that, after 24 h of co‐incubation, most nanoparticles are localized within the mitochondria, accompanied by near‐complete degradation of the 4s shell. In contrast, the group without TPP modification (Au_2_Pt@4sMSN/PS@CM) showed no mitochondrial internalization and only partial shell degradation, likely due to the presence of abundant GSH around the mitochondria,^[^ ^28^ ^]^ which accelerated shell degradation.

To confirm the intracellular mitochondrial localization of nanoparticles rather than superficial membrane adherence, serial ultrathin sectioning (70 nm thickness) was further imaged with Bio‐TEM. Correlative TEM micrographs of consecutive serial sections (Figure S13a,b, Supporting Information) displayed matching ultrastructural features within spatially aligned cellular domains (the corresponding black dashed rectangles), thereby confirming that they originated from the same cell. Figure 4d showed two nanoparticles within a single mitochondrion (red dashed rectangle named 1), whereas only one nanoparticle was observed in Figure 4e (red dashed rectangle named 1.1). This phenomenon arose from the 3D architecture of the biological specimen, where the two nanoparticles were located at different positions along the z‐axis within the mitochondrion. During progressive sectioning, the upper nanoparticle located at a higher axial position was physically separated, leaving the lower particle visible in subsequent sections (a sectioned fragment of the nanoparticle). Therefore, these findings demonstrate that Au_2_Pt@4sMSN/PS‐TPP@CM can be internalized into mitochondria, rather than merely adhering to the mitochondrial membrane surface. While previous studies^[^ ^29^, ^30^, ^31^, ^32^, ^33^ ^]^ have demonstrated that TPP modification can guide nanoparticles to the mitochondrial membrane periphery or matrix (small‐sized nanoparticles), this study provided the visualized evidence of nanoparticles larger than 100 nm that were internalized into mitochondria.

Typically, larger TPP‐modified nanoparticles cannot enter mitochondria without external forces. However, in our designed nanoplatform, the tetrasulfide bond‐bridged shell can chemically react with GSH in physiological environments to produce H_2_S, thereby generating a chemical driving force. To validate this mechanism, we further investigated nanoparticle motility behaviors under varying GSH concentrations, using Au_2_Pt@MSN (silica nanoparticles without tetrasulfide bonds) as a control. As shown in Figure 4f, the enhanced diffusion of Au_2_Pt@4sMSN displayed a clear dependence on GSH concentration (0, 2, 10, 50 mm). In PBS solution, Au_2_Pt@4sMSN exhibited typical Brownian motion. However, increasing the GSH concentration resulted in a significant extension of its trajectories. In contrast, Au_2_Pt@MSN maintained consistently low diffusive behavior regardless of GSH presence (Figure 4g). Mean square displacement (MSD) analysis further revealed that Au_2_Pt@4sMSN exhibited a linear MSD increase over time (Figure 4h), with diffusion coefficients positively correlating with GSH concentrations (Figure 4j). These results confirm that the enhanced diffusion of Au_2_Pt@4sMSN nanoparticles is directly triggered by GSH‐induced chemical propulsion. Conversely, the MSD curve and diffusion coefficients of Au_2_Pt@MSN remained relatively unchanged (Figure 4i,k), further confirming its inertness and insensitivity to the GSH‐rich environment.

Collectively, these findings provide insight into the underlying mechanism of mitochondrial internalization. Specifically, as shown in Figure 4l, nanoparticles first approach or adhere to mitochondria guided by TPP targeting. Subsequently, the high local concentration of GSH near mitochondria induces rapid degradation of the 4S silica shell, releasing H_2_S. This chemical reaction generates propulsion forces that enhance autonomous nanoparticle diffusion toward mitochondria. Simultaneously, the mitochondrial membrane potential gradient further facilitates nanoparticle uptake. Moreover, upon contact with mitochondrial membranes, the nanoparticle surfaces interface directly with mitochondria and are shielded from the GSH reaction, creating an asymmetric degradation pattern. This structural asymmetry ensures selective H_2_S generation on surfaces opposite the mitochondrial interface, further promoting directional diffusion and mitochondrial internalization. Indeed, as illustrated in Figure 1m, Au_2_Pt@4sMSN degradation also occurs asymmetrically in normal GSH exposure, reinforcing the formation of Janus structures, thereby significantly enhancing autonomous motility and improving tumor penetration.

### 2.7 Mechanism Investigation of H_2_S‐Driving Enhanced EDT, PDT, and mPTT

**Figure 5:**
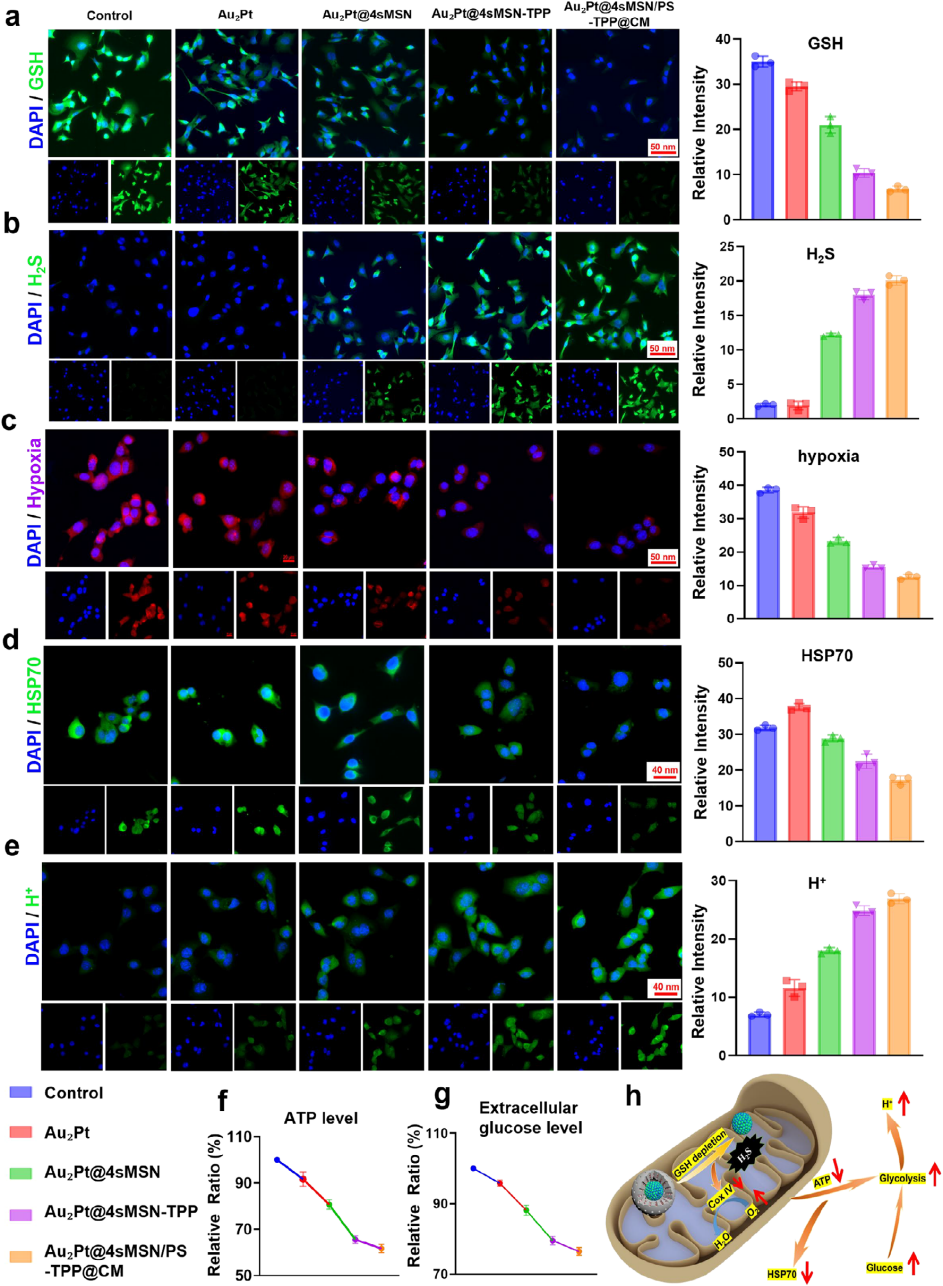
Mechanism investigation of H_2_S‐driving enhanced CDT, PDT, and mPTT. a) Fluorescence images and analysis of ThiolTracker Violet staining. b) Fluorescence images and analysis of WSP‐5 probe staining. c) Fluorescence images and analysis of Ru(dpp)_3_Cl_2_ probe staining. d) Fluorescence images and analysis of HSP70 expression. e) Fluorescence images and analysis of BCECF‐AM probe staining. f) Quantitative analysis of ATP level. g) Quantitative analysis of extracellular glucose concentration. h) Schematic diagram of H_2_S‐driven metabolic reprogramming activating molecular cues to enhance PDT, mPTT, and EDT efficacy.

We next explored the role of H_2_S generated by Au_2_Pt@4sMSN/PS‐TPP@CM in enhancing EDT, PDT, and mPTT at the cellular level. **Figure** **5**a displays fluorescence images of the GSH‐responsive degradation of Au_2_Pt@4sMSN/PS‐TPP@CM within Hepa1‐6 cells. Cells without any treatment exhibited the highest levels of intracellular GSH. In contrast, co‐incubation with Au_2_Pt@4sMSN led to a significant depletion of GSH, which was further decreased by treating with mitochondria‐targeted Au_2_Pt@4sMSN‐TPP. It was attributed to the higher concentration of GSH in the mitochondrial microenvironment, leading to more rapid consumption during the same incubation period. These results were further corroborated by Figure 5b. The Au_2_Pt@4sMSN group‐treated cells exhibited H_2_S generation, while TPP‐modified nanoparticles, Au_2_Pt@4sMSN‐TPP, resulted in greater H_2_S production under the same reaction conditions. Moreover, the CM‐coated nanoparticles Au_2_Pt@4sMSN/PS‐TPP@CM exhibited a further slight increase in H_2_S generation due to enhanced cellular uptake efficiency.

H_2_S has been widely reported to compete with oxygen by binding to cytochrome c oxidase (Complex IV) in mitochondria, thereby blocking electron transport and ultimately inhibiting oxidative phosphorylation (OXPHOS).^[^ ^34^, ^35^ ^]^ Consequently, the precise mitochondria‐targeted H_2_S generation could further amplify this cytotoxic effect. As shown in Figure 5c, the impact of H_2_S‐mediated mitochondrial respiration inhibition was validated by intracellular oxygen concentration measurements, which showed significantly higher oxygen levels in the Au_2_Pt@4sMSN/PS‐TPP@CM treated group compared to the control group. This result suggests a reduction in mitochondrial oxygen consumption, indicating the OXPHOS inhibition. Notably, previous studies have shown that the H_2_S‐mediated suppression of maximal oxygen consumption rate (OCR) persisted for 24–48 h, demonstrating a “metabolic memory” effect.^[^ ^16^ ^]^ It means that H_2_S elimination failed to restore cellular maximal OCR, resulting in alleviated cellular hypoxia and the establishment of a favorable microenvironment for enhanced PDT. In addition, the Au_2_Pt group exhibited slight alleviation of hypoxia, attributed to its CAT‐like activity in decomposing the abundant H_2_O_2_ in TME to generate O_2_.

As a direct consequence of OXPHOS inhibition, mitochondrial function was impaired, leading to a decrease in ATP production, as shown in Figure 5f. This ATP depletion was accompanied by a reduction in HSP70 expression (Figure 5d), a key molecular chaperone involved in resistance to mPTT. Furthermore, the disruption of mitochondrial energy metabolism forced tumor cells to rely on hyperactive glycolysis to compensate for impaired ATP production.^[^ ^36^ ^]^ This metabolic reprogramming was evidenced by increased intracellular acidity (Figure 5e), indicating elevated lactate production, a hallmark of enhanced glycolytic activity.^[^ ^37^ ^]^ Reduced extracellular glucose levels (Figure 5g) further confirmed increased glucose uptake as a compensatory response to mitochondrial dysfunction.

Taken together, these findings collectively validate that the Au_2_Pt@4sMSN/PS‐TPP@CM mediated targeted H_2_S release effectively disrupts mitochondrial function and hyperactivates glycolytic metabolic pathways. As illustrated in Figure 5h, the release of H_2_S, as a gasotransmitter, triggers a cascade of interconnected biochemical events that synergistically enhance PDT, mPTT, and CDT: 1) H_2_S‐induced GSH depletion weakens the tumor's antioxidant defense, sensitizing cancer cells to oxidative stress. 2) A reduction in oxygen consumption increases intracellular oxygen availability, enhancing PDT efficacy. 3) ATP depletion and HSP70 downregulation suppress thermotolerance, making tumor cells more sensitive to mPTT. 4) Hyperactivated glycolysis leads to increased glucose uptake and a decrease in intracellular pH, which act as substrates to promote the EDT of the Au_2_Pt nanozyme core, enhancing Fenton‐like reactions. Therefore, H_2_S serves as the primary driving cue, initiating a domino‐like cascade that integrates PDT, mPTT, and CDT into a synergistic therapeutic nanoplatform, significantly amplifying tumor cell vulnerability to oxidative stress and therapeutic interventions.

### 2.8 H_2_S‐Driven Multimodal Cascade Therapy In Vitro

It was demonstrated that Au_2_Pt@4sMSN/PS‐TPP@CM‐mediated H_2_S gas therapy triggered a cascade of biological events enhancing mPTT, PDT, and EDT efficacy. Building upon this, we systematically investigated its anti‐tumor efficacy and underlying mechanisms. Seven groups were included in these cell experiments: PBS, Au_2_Pt, Au_2_Pt@4sMSN, Au_2_Pt@4sMSN/PS‐TPP@CM, Au_2_Pt@4sMSN/PS‐TPP@CM + L1, Au_2_Pt@4sMSN/PS‐TPP@CM + L2, and Au_2_Pt@4sMSN/PS‐TPP@CM + L1 + L2.

**Figure 6:**
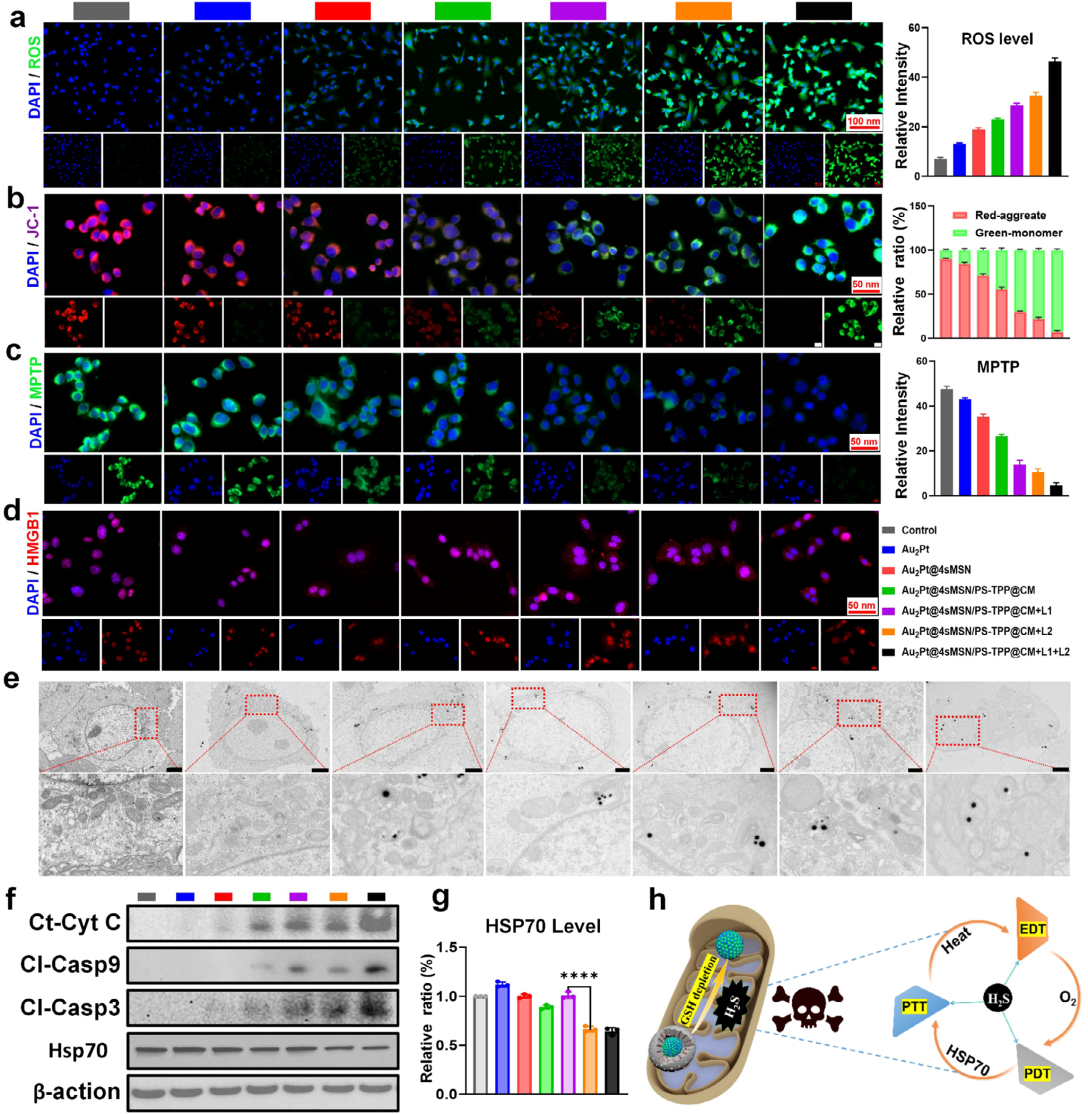
H_2_S‐driven multimodal cascade therapy in vitro. a) Fluorescence images and analysis of ROS staining. b) Fluorescence images and analysis of JC‐1 staining. c) Fluorescence images and analysis of MPTP detection. d) Fluorescence images and analysis of HMGB1 expression. e) Bio‐TEM images analysis of mitochondria morphology. The bar is 2 µm. f) Western Blot analysis of the expression of mitochondrial apoptosis‐related proteins. g) Quantitative analysis of HSP70 expression. h) Schematic diagram of an H_2_S‐driven self‐amplifying multimodal therapeutic crosstalk network.

As shown in **Figure** **6**a, ROS fluorescence intensities in Hepa1‐6 cells under various treatments were quantified. The CDT driven by Au_2_Pt nanozymes under acidic conditions induced negligible ROS generation. However, upon the introduction of the 4sMSN shell, intracellular GSH was significantly depleted, thereby weakening the antioxidant defense of tumor cells and resulting in a high level of ROS generation. Notably, H_2_S generated in situ further increased ROS levels in Au_2_Pt@4sMSN/PS‐TPP@CM group. This could be attributed to a dual mechanism: mitochondrial targeting enhanced localized oxidative stress, while H_2_S‐stimulated glycolytic hyperactivity triggered intracellular acidification, thereby augmenting the POD‐like activity of Au_2_Pt. Concurrently, the accelerated glucose uptake supplied abundant substrate for GOx‐like activity, thereby exacerbating intracellular acidification and creating a self‐amplifying acidic microenvironment for CDT. In addition, the application of mPTT or PDT individually led to a further increase in ROS levels, while combining both modalities resulted in maximal ROS production.

Given that increased intracellular pH ROS levels only serve as an indirect indicator of mitochondrial status, we then comprehensively assessed mitochondrial function after various treatments. As demonstrated in Figure 6b, JC‐1 staining revealed a stepwise reduction in red fluorescence intensity (JC‐1 aggregates) with a corresponding increase in green fluorescence (JC‐1 monomers) upon sequential addition of treatment components, indicative of progressive mitochondrial membrane potential depolarization. Complete collapse of membrane potential occurred in the Au_2_Pt@4sMSN/PS‐TPP@CM + L1 + L2 group, evidenced by near‐total red fluorescence disappearance. Complementary MPTP imaging results (Figure 6c) confirmed gradual MPTP opening with the diminishing mitochondrial green fluorescence staining. The mitochondrial damage was further visualized by bio‐electron microscopy (Figure 6e). In the control group, mitochondria exhibited intact double membranes and well‐defined cristae, indicating normal mitochondrial morphology and function. Following mPTT or PDT, structural damage to the mitochondrial membrane was observed, accompanied by swelling of the cristae. Notably, in the combination therapy group, the mitochondria were severely compromised, with complete loss of cristae and outer membrane integrity.

Additionally, PDT and mPTT also synergistically elicited immunogenic cell death (ICD) and potentiating antitumor immunity. As shown in Figure S14 (Supporting Information), individual PDT or mPTT treatment significantly increased the expression of CRT, while the combined therapy further enhanced CRT upregulation. Moreover, High mobility group box 1 (HMGB1) gradually translocated from the nucleus to the cytoplasm, culminating in extensive cytoplasmic dispersion in the combined treatment group (Figure 6d). In addition, the mPTT effect was less effective than PDT, which may be attributed to the protective response mediated by the heat shock protein family.^[^ ^38^ ^]^

Excessive ROS levels and mitochondrial dysfunction often trigger the activation of intrinsic apoptotic pathways. As shown in Figure 6f, the increased intracellular cytochrome C levels confirmed substantial release of this apoptogenic factor from mitochondria, marking the initiation of mitochondria‐dependent apoptotic signaling. The upregulation of downstream cleaved caspase‐9 and cleaved caspase‐3 further validated that Au_2_Pt@4sMSN/PS‐TPP@CM induces Hepa1‐6 cell apoptosis primarily through the intrinsic mitochondrial pathway.

Collectively, these results indicate that Au_2_Pt@4sMSN/PS‐TPP@CM enhances oxidative stress, disrupts mitochondrial function, and activates both intrinsic apoptosis pathways and ICD, culminating in robust antitumor effects. Notably, the combination therapy exhibited superior efficacy compared to laser monotherapy across impairing mitochondrial function, pro‐apoptotic signaling, and ICD induction. This enhancement can be attributed to a self‐reinforcing positive feedback circuit integrating PDT, mPTT, and EDT. As illustrated in Figure 6h, mPTT‐generated mild hyperthermia amplified the multienzyme cascade activity of Au_2_Pt, substantially increasing •OH and O_2_ (confirmed in Figure 2h,j). The resultant oxygen pool sustains PDT efficacy, while mitochondria‐targeted PDT further disrupts mitochondrial respiration, leading to pronounced downregulation of ATP‐dependent HSP70 expression. This mitochondria‐targeted modality also impairs HSP70 activity through ROS‐mediated oxidative post‐translational modifications^[^ ^39^ ^]^ and enhances mitochondrial thermal sensitivity,^[^ ^40^ ^]^ synergistically amplifying mPTT efficacy. As demonstrated in Figure S15 (Supporting Information), cellular ATP levels were significantly lower following PDT compared to mPTT treatment. Concurrently, HSP70 expression showed a slight upregulation following mPTT, whereas a marked HSP70 expression downregulation was observed after PDT (Figure 6g). This was attributed to the chaperone activity of HSP family members is significantly impaired when intracellular ATP levels drop below a critical threshold, as their function depends on ATP‐driven protein folding machinery.^[^ ^41^ ^]^ This ultimately results in reduced cellular thermotolerance. Therefore, this mitochondria‐targeted nanoplatform strategically integrates CDT, mPTT, and PDT within an H_2_S‐driven positive feedback loop, representing a promising therapeutic paradigm for maximizing synergistic tumor eradication.

### 2.9 Biodistribution and Tumor‐Targeting Assessment In Vivo

**Figure 7:**
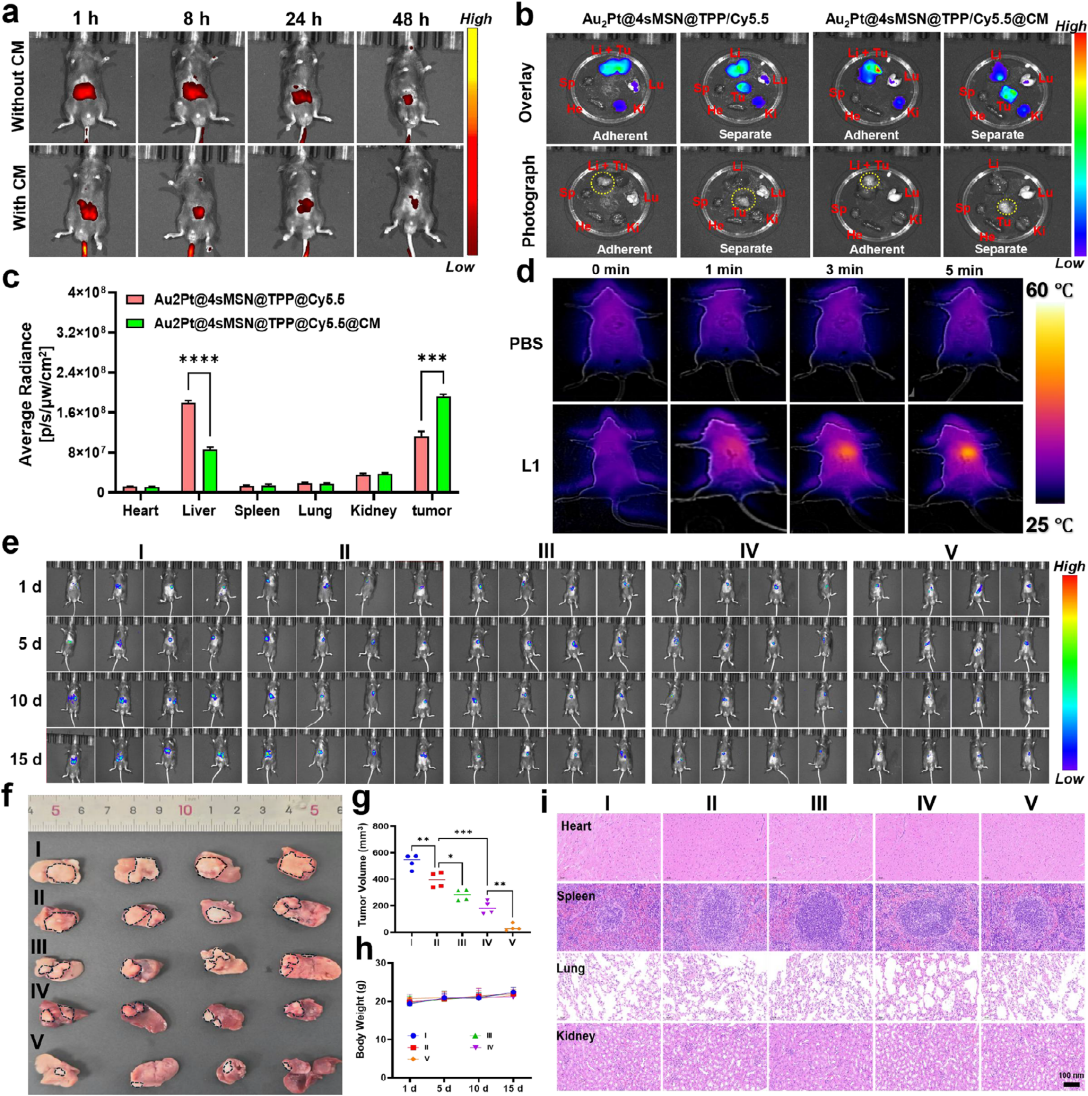
The evaluation of biodistribution and anti‐tumor efficiency in vivo. a) In vivo biodistribution fluorescence images of Au_2_Pt@4sMSN/Cy5.5‐TPP and Au_2_Pt@4sMSN/Cy5.5‐TPP@CM at different time points. b) Fluorescence imaging and c) Quantitative analysis of signals in excised organs using an in vivo imaging system. d) Real‐time infrared thermal images of mice injected with PBS or nanoparticles under 808 nm laser irradiation. e) Representative bioluminescence images acquired on days 1, 5,10, and 15. f) Photographs of excised tumors and g) corresponding tumor volume quantification. h) Body weight change curve of mice during treatment. i) H&E staining of major organ tissues.

To investigate the in vivo biodistribution and tumor‐targeting efficiency of the nanoparticles, mice were randomly divided into two groups (Without CM group and With CM group): Au_2_Pt@4sMSN/Cy5.5‐TPP and Au_2_Pt@4sMSN/Cy5.5‐TPP@CM. As shown in **Figure** **7**a, the real‐time fluorescence images at different time points post‐intravenous administration showed that the nanoparticle signal peaked at ≈1 h, gradually declined over time, but remained detectable up to 48 h. Notably, the fluorescence intensity in the CM‐modified group was significantly higher than that in the non‐CM group at 48 h, suggesting enhanced accumulation and prolonged retention at the tumor site of Au_2_Pt@4sMSN/Cy5.5‐TPP@CM. Considering that nanoparticles are predominantly metabolized by the liver,^[^ ^42^ ^]^ these results were insufficient to distinguish between accumulation in the tumor and that in normal liver tissue. Thus, mice were euthanized 12 h post‐injection, and major organs (heart, liver, spleen, lung, and kidney) were harvested for IVIS imaging (Figure 7b). The results confirmed predominant hepatic accumulation in both groups, with markedly lower fluorescence intensity detected in the kidneys. In addition, tumor tissue was separated from adjacent normal hepatic tissue for further imaging. It was found that the CM‐targeted group exhibited markedly higher fluorescence in the tumor region, while the non‐targeted group showed no significant difference between tumor and normal tissue. Quantitative fluorescence analysis (Figure 7c) revealed a significantly enhanced tumor‐targeting capacity in the CM group compared to the non‐CM group.

### 2.10 Photothermal Performance In Vivo

Based on biodistribution results, the CM group was selected for subsequent photothermal effect assessment and therapeutic evaluation. Mice were divided into PBS and Au_2_Pt@4sMSN/PS‐TPP@CM groups. 12 h after tail vein injection of nanoparticles, the upper abdominal region of mice was exposed to 808 nm laser irradiation for 5 min. Infrared thermal imaging revealed that the PBS group displayed no temperature change, whereas the nanoparticle‐treated group exhibited a rapid temperature rise, reaching ≈60 °C within 5 min (Figure 7d). This result demonstrates the efficient photothermal conversion of Au_2_Pt@4sMSN/PS‐TPP@CM in vivo, highlighting its potential for photothermal therapy applications.

### 2.11 In Vivo Anti‐Tumor Treatment

Inspired by the therapeutic potential observed in vitro, we further established an orthotopic liver tumor model to evaluate the in vivo anti‐tumor efficacy of the nanoplatform, in which luciferin‐based bioluminescence in vivo imaging was employed to dynamically monitor tumor progression. The mice were randomly divided into five groups: I) PBS; II) Au_2_Pt@4sMSN/PS‐TPP@CM; III) Au_2_Pt@4sMSN/PS‐TPP@CM + L1; IV) Au_2_Pt@4sMSN/PS‐TPP@CM + L2; V) Au_2_Pt@4sMSN/PS‐TPP@CM + L1 + L2. As shown in Figure 7e, bioluminescence imaging revealed rapid tumor progression in the control group. In mice treated with Au_2_Pt@4sMSN/PS‐TPP@CM, tumor growth was moderately attenuated, likely attributable to the synergistic effects of H_2_S‐mediated GT and Au_2_Pt nanozyme‐catalyzed EDT. The Au_2_Pt@4sMSN/PS‐TPP@CM + L2 group exhibited a gradual decline in bioluminescence signal intensity during treatment, accompanied by significant tumor volume reduction. In contrast, the Au_2_Pt@4sMSN/PS‐TPP@CM + L1 group showed slower tumor progression due to the limited therapeutic effect of mPTT. Notably, the combination group (Au_2_Pt@4sMSN/PS‐TPP@CM + L1 + L2) demonstrated superior tumor suppression compared to either monotherapy (Au_2_Pt@4sMSN/PS‐TPP@CM + L1 or Au_2_Pt@4sMSN/PS‐TPP@CM + L2), suggesting H_2_S‐mediated potent synergistic efficacy across PDT, EDT, and mPTT. Furthermore, the representative orthotopic liver tumor images on day 15 (Figure 7f) showed that the tumor was nearly completely eradicated in the final treatment group (tumor margins demarcated by black dashed lines), exhibiting a remarkable tumor shrinkage of over 90% (Figure 7g). These images correlated with bioluminescence data, further confirming the therapeutic superiority of Au_2_Pt@4sMSN/PS‐TPP@CM in orthotopic liver cancer treatment through multimodal therapeutic integration.

The body weight of mice was also closely monitored throughout the treatment period, with no significant differences observed between treatments and control groups, suggesting no apparent adverse effects on the overall health status of the animals (Figure 7h). In addition, histological analysis of major organs using Hematoxylin and eosin (H&E) staining was conducted to evaluate potential tissue damage or pathological changes (Figure 7i), which revealed normal tissue morphology and architecture across all examined organs. Together, these results demonstrate that Au_2_Pt@4sMSN/PS‐TPP@CM has favorable biosafety and biocompatibility.

**Figure 8:**
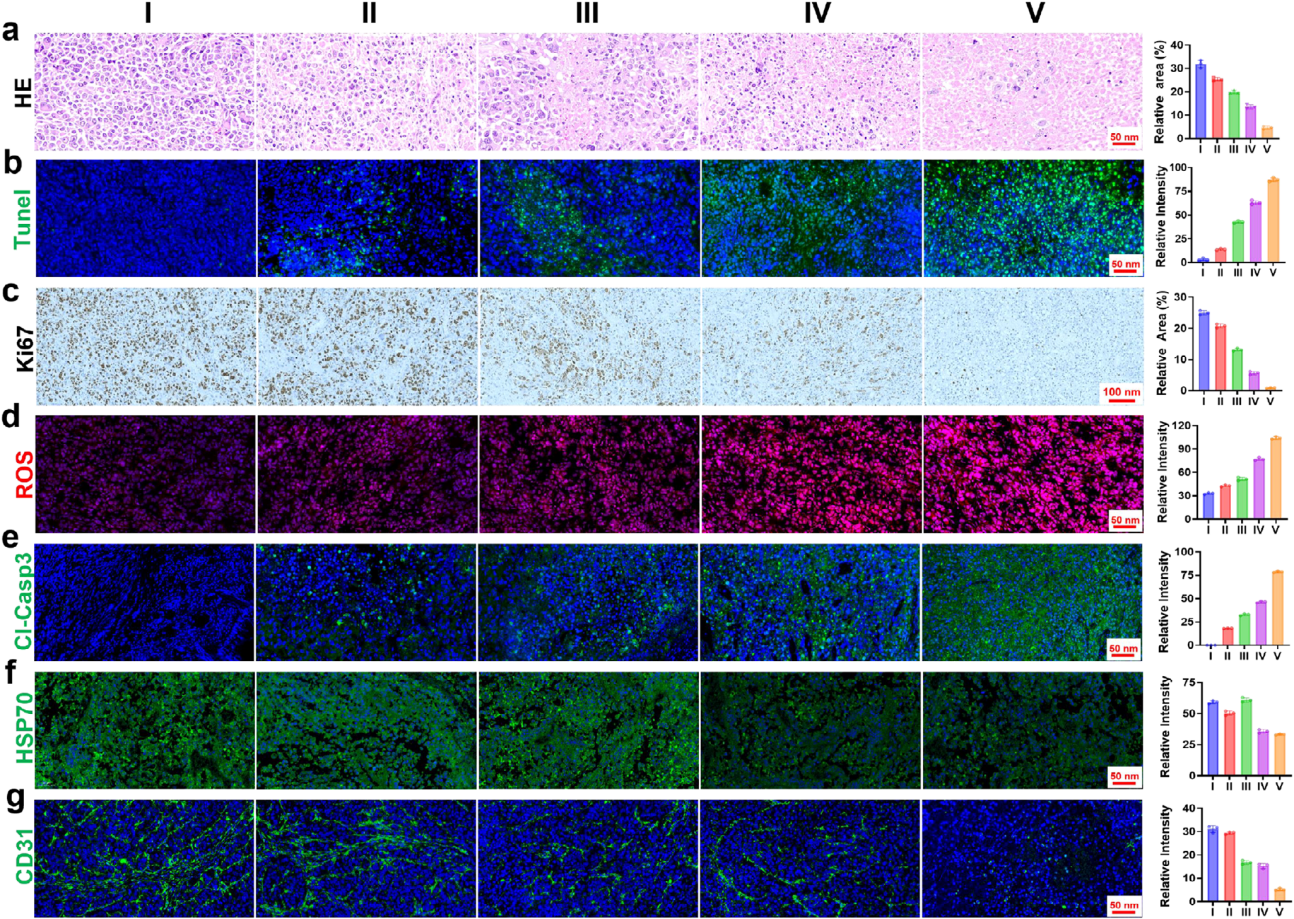
The histopathological evaluations in vivo. a) H&E staining of tumor tissues from different groups. b) Representative fluorescence images and analysis of TUNEL staining. c) Immunohistochemical analysis of Ki‐67 expression in tumors from different groups. d) ROS fluorescence imaging in tumors from different groups. e) Immunostaining of cleaved caspase‐3 in different groups. f) HSP70 protein expression level in different groups. g) CD31 immunostaining of tumor tissues with different treatments.

To systematically elucidate the antitumor mechanism of Au_2_Pt@4sMSN/PS‐TPP@CM in vivo, multidimensional histopathological evaluations were performed on tumor tissues. H&E staining revealed characteristic histoarchitectural features of Hepa1‐6 tumors in control groups, including densely packed pleomorphic nuclei, frequent mitotic activity, and extensive neovascularization (**Figure** **8**a), consistent with their aggressive proliferative and invasive phenotypes. In contrast, therapeutic groups exhibited apoptosis induction, with the combination therapy group (Group V) displaying pronounced morphological hallmarks of apoptosis, such as widespread nuclear pyknosis, karyorrhexis, and cytoplasmic vacuolization. This result was further confirmed by TUNEL fluorescence staining (Figure 8b) and Ki‐67 (Figure 8c) immunohistochemical analysis, which collectively demonstrated that the nanoplatform effectively induced apoptosis and suppressed proliferation, thereby exhibiting potent antitumor efficacy.

ROS fluorescence imaging (Figure 8d) revealed a significant difference between monotherapy and combination treatment, with monotherapy inducing moderate oxidative stress, while the combination group triggered a substantial increase in ROS levels. In addition, mitochondrial apoptosis pathway activation was corroborated by cleaved caspase‐3 immunostaining (Figure 8e). The above results indicated that mPTT or PDT exhibited moderate tumor inhibition, whereas their combination led to a marked therapeutic improvement. Furthermore, HSP70 staining (Figure 8f) uncovered the critical therapeutic synergies: mPTT monotherapy upregulated HSP70 expression, indicating a compensatory heat‐shock adaptation, whereas PDT monotherapy suppressed HSP70 levels. This finding suggested that mitochondria‐targeting PDT sensitizes tumors to mPTT by disrupting heat‐shock defenses, thus achieving a synergistic therapeutic feedback loop.

The Hepa1‐6 tumor model is characterized by prominent hypervascularization, which facilitates rapid tumor progression through sustained nutrient supply. CD31 immunofluorescence staining (Figure 8g) revealed a stark contrast in vascular architecture between control tumors exhibiting densely organized vascular networks and the combination treatment group showing complete ablation of tumor‐associated vasculature. This structural collapse of the vascular framework confirmed that the nanoplatform could effectively suppress growth by inhibiting nutrient supply.

Immunofluorescence of CD4⁺ and CD8⁺ T cells (Figure S16, Supporting Information) revealed minimal baseline infiltration in untreated tumors. Following treatment, particularly in the combination group, intertumoral CD4⁺ and CD8⁺ T‐cell infiltration increased markedly. These findings are consistent with the cooperative effects of the multimodal regimen and highlight the platform's potential to enhance anti‐tumor immunity.

In summary, following tumor cell‐specific targeting, the nanoplatform synergistically depletes GSH while enabling mitochondria‐targeted H_2_S delivery. The released H_2_S exerts three synergistic effects: 1) It inhibits oxygen electron transport, alleviating tumor hypoxia to enhance PDT. 2) It decreases ATP production and reduces HSP70, thereby sensitizing cancer cells to mild PTT. 3) It hyperactivates glycolysis, accelerating glucose uptake and exacerbating intracellular acidity, which provides abundant substrates (glucose, H^+^, and H_2_O_2_) for the enzymatic activities (POD, CAT, GOx) of Au_2_Pt, thus boosting EDT. Moreover, these augmented modalities engage in a self‐sustaining feedback circuit: PDT‐induced hyperthermia potentiates EDT's catalytic activity, driving oxygen generation (via the CAT‐like activity of Au_2_Pt core) to maintain PDT. Subsequently, the mitochondrial‐localized PDT disrupts ATP‐dependent HSP70 synthesis and activity, in turn amplifying PTT sensitivity. Ultimately, these mutually reinforcing events result in mitochondrial apoptosis, significantly inhibiting the growth of orthotopic hepatocellular carcinoma.

Overall, this nanoplatform, guided by the principle of programming on‐demand molecular crosstalk at the molecular level, lays a solid foundation for the development of self‐reinforcing, multimodal nanotherapeutics. Given that GT, PDT, and mPTT within this platform can trigger ICD alongside apoptosis, future work will systematically interrogate immune memory in tumor‐rechallenge and metastasis models and evaluate combination strategies with immune‐checkpoint blockade to potentiate durable, systemic antitumor immunity. Building on the potent therapeutic cascade, integration with immunotherapy is expected to further extend this paradigm and enhance its clinical translation potential, thereby providing meaningful avenues to overcome the limitations of conventional combination therapy.

## 3 Conclusion

In conclusion, this mitochondria‐targeting nanoplatform, Au_2_Pt@4sMSN/PS‐TPP@CM, integrates GT, EDT, PDT, and mPTT into a multifunctional “four‐in‐one system” for self‐cascading multimodal cancer therapy. It enables mitochondria‐targeted delivery of H_2_S to hyperactive glycolysis, thereby generating biochemical cues that synergistically enhance the therapeutic efficacy of PDT, mPTT, and EDT. These coordinated modalities further establish a self‐reinforcing therapeutic circuit through molecular crosstalk, leading to efficient eradication of orthotopic hepatocellular carcinoma. Notably, we provide direct Bio‐TEM evidence of mitochondrial internalization of >100 nm nanoparticles—a finding that suggests new possibilities for subcellular‐targeted nanomedicines. Collectively, this study presents a mitochondria‐targeting therapeutic strategy that orchestrates self‐amplifying multimodal coordination via gasotransmitter‐mediated metabolic crosstalk, offering a novel conceptual framework for developing mechanism‐driven combination therapies.

## Conflict of Interest

The authors declare no conflict of interest.

## Author Contributions

C.L., X.M., and S.F. contributed equally to this work. C.L. performed most of the experiments and manuscript writing. X.M. supported the material design, while S.F. and B.C. assisted with the animal experiments. X.W., L.H., X.Y., and Y.L. contributed to parts of the cell‐based experiments. J.M.R. revised the manuscript. Z.Z., J.J., and H.Z. jointly supervised, founded, and reviewed this work.

## Supporting information



Supporting Information

## Data Availability

The data that support the findings of this study are available from the corresponding author upon reasonable request.
