# Supplementary material for: Mitochondria‐Targeted Nanomotor: H2S‐Driven Cascade Therapy for Hepatocellular Carcinoma
Source: Adv Mater. 2025 Dec 1;38(8):e13757. doi: 10.1002/adma.202513757 (PMC12879285; doi:10.1002/adma.202513757)
Supplement: Supplementary file 1 — Supporting Information [file ADMA-38-e13757-s001.pdf]

ADVANCED MATERIALS

Supporting Information

for *Adv. Mater.*, DOI 10.1002/adma.202513757

Mitochondria-Targeted Nanomotor: H_2S-Driven Cascade Therapy for Hepatocellular Carcinoma

Chengcheng Li, Xiaodong Ma, Shiji Fang, Biao Chen, Xinru Wang, Lingyun He, Xin Yang, Yuanqiang Li, Jessica M. Rosenholm, Zhongwei Zhao, Jiansong Ji* and Hongbo Zhang**

ADVANCED MATERIALS

Supporting Information

for *Adv. Mater.*, DOI 10.1002/adma.202513757

Mitochondria-Targeted Nanomotor: H_2S-Driven Cascade Therapy for Hepatocellular Carcinoma

Chengcheng Li, Xiaodong Ma, Shiji Fang, Biao Chen, Xinru Wang, Lingyun He, Xin Yang, Yuanqiang Li, Jessica M. Rosenholm, Zhongwei Zhao, Jiansong Ji* and Hongbo Zhang**

Supporting Information

Mitochondria-Targeted Nanomotor: H_2S-Driven Cascade Therapy for Hepatocellular Carcinoma

Chengcheng Li†, Xiaodong Ma†, Shiji Fang†, Biao Chen, Xinru Wang, Lingyun He, Xin Yang, Yuanqiang Li, Jessica M. Rosenholm, Zhongwei Zhao*, Jiansong Ji*, Hongbo Zhang*

C. Li, S. Fang, B. Chen, Z. Zhao, J. Ji, H. Zhang
Zhejiang Key Laboratory of Imaging and Interventional Medicine, Zhejiang Engineering Research Center of Interventional Medicine Engineering and Biotechnology, Department of Radiology, Lishui Central Hospital, The Fifth Affiliated Hospital of Wenzhou Medical University, Lishui 323000, China
E-mail: zhaozhongwei@zju.edu.cn, jijiansong@zju.edu.cn

X. Ma, X. Wang, L. He, X. Yang, H. Zhang
Joint Centre of Translational Medicine, Wenzhou Key Laboratory of Interdiscipline and Translational Medicine, the First Affiliated Hospital of Wenzhou Medical University, 325000, Wenzhou, China
E-mail: hongbo.zhang@abo.fi

C. Li, X. Ma, Y. Li, J.M. Rosenholm, H. Zhang
Pharmaceutical Sciences Laboratory, Faculty of Science and Engineering, Åbo Akademi University, 20500, Turku, Finland

H. Zhang
Turku Bioscience Centre, University of Turku and Åbo Akademi University, 20520, Turku, Finland

†These authors contributed equally to this work.
*Corresponding author.

Materials and Methods

Materials

All the chemicals were of analytical grade and used without further purification. Gold chloride trihydrate ($HAuCl_4$), Chloroplatinic acid hexahydrate (H_2PtCl_6), ascorbic acid, hexadecyltrimethylammonium bromide (CTAB), bis[3-(triethoxysily)propyl] tetrasulfide (BTES), methyl alcohol, tetraethyl orthosilicate (TEOS), 5,10,15,20-tetrakis (4-hydroxyphenyl)-21H (THPP), 5,5'-Dithiobis-(2-Nitrobenzoic Acid) (DTNB), Dimethyl sulfoxide (DMSO), NHS (N-Hydroxysuccinimide), (3-Aminopropyl) triethoxysilane (APTES), ammonium nitrate (NH_4NO_3), (3-Carboxypropyl)triphenylphosphonium bromide (TPP-COOH), EDC (1-Ethyl-3-(3-dimethyl aminopropyl) carbodiimide), DMF (N, N-Dimethylformamide), and L-Glutathione Reduced (GSH) were purchased from Sigma-Aldrich (USA). BCECF AM (2',7'-bis-(2-carboxyethyl)-5-(and-6)-carboxyfluorescein, acetoxymethyl ester), and WSP-5 were purchased from MedChem Express (USA). Cleaved-caspase 9, HSP70, Cytochrome C, Cleaved-caspase 3, CRT, and HMGB1 antibodies were obtained from Proteintech (Wuhan, China). Near infrared (NIR) 808nm laser and 606nm laser were obtained from Xi'an Lazer Electronic Technology Co., Ltd. (China).

Characterization

Particle size and Zeta potential were determined using Zetasizer Nano (Malvern Panalytical, UK). The morphology structure of nanoparticles was imaged using a transmission electron microscope JEM-1400 Plus TEM (JEOL, Japan). BD FACSFortessa™ (BD Biosciences, USA) was used for cell flow cytometry. Zeiss LSM 880 with Airyscan confocal microscope (Zeiss, Germany) and an Inverted fluorescence microscope (Axio Observer, Zeiss) were used for living cell imaging in all cell experiments. Ultraviolet-visible absorption spectra were tested using a SpectraMax Plus spectrophotometer (Molecular Devices, USA). The crystallographic structure was analyzed using a Bruker D8 Advance X-ray diffractometer (Bruker, Germany). Surface chemical analysis was conducted using a Thermo Scientific ESCALAB Xi^+ (Thermo Fisher Scientific, USA) and a Fourier transform infrared (FTIR) spectrometer (PerkinElmer, USA).

Synthesis of Au_2Pt Nanozyme

The Au_2Pt nanoparticles were prepared using the previous method. [1] Briefly, 23.5698 mg $HAuCl_4$, 31.074 mg H_2PtCl_6, and 57.4 mg L-proline were dispersed in 20 mL of deionized (DI) water. Then the pH value of the mixture solution was adjusted to 10 by adding 0.1 mmol L^{-1} NaOH solution. Subsequently, 20 mmol/L ascorbic acid was added at 500 r/min stirring. Finally, the Au_2Pt

nanoparticles were collected with 12000 g/min centrifuging after reacting for 15 min at room temperature.

Synthesis of Au_2Pt @4sMSN and Au_2Pt @MSN

The collected Au_2Pt nanoparticles were resuspended in 50 mL of deionized water. After adding 100 μL CTAB (0.5 mol L^{-1}), the pH value of the solution was adjusted to 10 with the addition of NaOH solution. Subsequently, 200 μL of methyl alcohol containing 40 μL of TEOS and 30 μL of BTES were added drop by drop. Au_2Pt@4sMSN nanoparticles were collected with an overnight reaction at room temperature. Finally, Au_2Pt@4sMSN was washed with 0.6 wt% NH_4NO_3 ethanol solution for removing the CTAB. The synthesis of Au_2Pt@MSN was performed following the same protocol as that of Au_2Pt@4sMSN, except that no BTES was added during the process.

Synthesis of Au_2Pt@4sMSN/PS-TPP

To prepare Au_2Pt@4sMSN-NH_2, 1 mL of Au_2Pt@4sMSN (5 mg/mL) was combined with 500 μL APTES and stirred at room temperature overnight. Then 5 mg Au_2Pt@4sMSN-NH_2, 5 mg TPP-COOH, 1.34 mg NHS, and 2.23 mg EDC were dispersed in 10 mL DMF solution and stirred overnight at room temperature to obtain Au_2Pt@4sMSN-TPP. Similarly, Au_2Pt@4sMSN/PS-TPP were obtained by the overnight stirring of 5 mg Au_2Pt@4sMSN-TPP and 3 mg tetrahydroporphyrin in ethanol solution.

Synthesis of Au_2Pt@4sMSN/PS-TPP@CM and Au_2Pt@4sMSN/Cy5.5-TPP@CM

Briefly, the Hepa 1-6 cellular membrane was isolated and purified following the previously established method. [2] Then, 5 mg Au_2Pt@4sMSN/PS-TPP and 7.5 mg freeze-dried cellular membrane were dispersed in DI water and stirred at 4 °C overnight. The resulting Au_2Pt@4sMSN/PS-TPP@CM were washed three times and then stored at 4 °C for reserved for future use. The synthesis procedure for Au_2Pt@4sMSN/Cy5.5-TPP@CM follows the same protocol as that of Au_2Pt@4sMSN/PS-TPP@CM, with the only modification being the replacement of PS with Cy5.5.

***In Vitro* Glutathione Responsiveness**

The GSH degradation was tested using a GSH Assay Kit (Yuanye, Shanghai) with the DTNB method. Briefly, different concentrations of Au_2Pt@4sMSN samples were co-incubated with 10 mM GSH solution at pH 6.5 for 4 hours, then mixed with DTNB solution and allowed to react at 25°C for 10 minutes. The supernatant was then collected and transferred to a 96-well plate for scanning the UV absorption peak at 412 nm. The morphology change was also imaged using TEM

after the different incubations at 0, 2, 8, 12, and 24 h time points. Similarly, H_2S generation was determined with the WSP-5 probe.

Multienzyme-mimicking activity

To simulate the intracellular response environment, Au_2Pt@4sMSN nanoparticles were co-incubated with 10 mM GSH at pH 6.5 for 24 h. Upon incubation, the tetrasulfide bond-bridged MSN undergoes cleavage, after which the resulting precipitate (Au_2Pt@WO) is collected and washed three times with H_2O_2 solution to thoroughly eliminate any residual GSH. The purified Au_2Pt@WO is then subjected to subsequent multienzyme-mimicking activity evaluations.

The POD-like activity was evaluated using the TMB method. Briefly, 30, 40, and 50 μl Au_2Pt@WO (2 mg/mL) were respectively mixed with HAc-NaAc buffer solution (0.1 M, pH 6.5) containing TMB (16 mM) and H_2O_2 (30 mM), and then collected appropriate reaction solution was collected for UV measurement. For the NIR 808 nm laser treatment group, the mixed solution was exposed to 808 nm NIR for 5 min. In addition, the POD-like activity was also assessed in HAc–NaAc buffer solutions at pH 7.4 and 6.5.

The CAT-like activity was analysed with the O_2 generation and H_2O_2 consumption. Briefly, 50 and 100 μl Au_2Pt@WO (2 mg/mL) and H_2O_2 solution (10 mM) were added into 5 mL DI water for 20 min. A dissolved oxygen meter (JPBJ-609L, Leici, China) was employed to measure the dissolved oxygen concentration, and the images of bubble generation in the mixed solution were captured using a camera.

The glucose depletion was measured by a Glucose Assay Kit with O-toluidine (Beyotine, Shanghai). O-toluidine reagent reacts with glucose to form a blue-green Schiff base with a maximum absorption wavelength of 630 nm. [3] Briefly, 100, 150, and 200 μg/mL Au_2Pt@WO were incubated with the 1 mg/mL glucose solution at 37 °C for 24h. For the NIR 808 nm laser treatment group, the mixture was irradiated with an 808 nm near-infrared laser for 10 minutes, and this procedure was repeated every 6 hours. Then, the solution was mixed with O-toluidine at 95 °C for 10 min and then cooled to 4 °C. Finally, the reaction mixture was transferred to a 96-well plate for the measurement of UV absorption wavelength at 630 nm. In addition, the pH value changes of the reaction solution as the gluconic acid generation were real-time monitored with a pH meter. A Hydrogen peroxide test kit (Beyotime, China) was used to determine the H_2O_2 generation, which determines the hydrogen peroxide concentration by oxidizing ferrous ions (Fe^{2+}) with hydrogen peroxide (H_2O_2) to produce ferric ions (Fe^{3+}), which then react with xylenol orange in a specific

solution to form a purple-coloured product. [4] 100, 150, and 200 μg/mL Au_2Pt@WO were incubated with the 1 mg/mL glucose solution at room temperature for 24h. After that, the ferrous ions were added to the reacting solution and then incubated for 30 minutes at 30 °C. Finally, 100 μL of the mixed solution was transferred to a 96-well plate for the measurement of UV absorption wavelength at 560 nm.

Photothermal Performance

To assess the photothermal performance of Au_2Pt@4sMSN, 0.1 mg/mL, 0.25 mg/mL, and 0.5 mg/mL Au_2Pt@4sMSN solutions were prepared in a 2 mL plastic tube. Each tube with a different concentration was irradiated for 5 minutes with an 808 nm laser at a power of 0.25 W, 0.5 W, and 1.0 W. DI water, the control group, was also exposed to the same laser for comparison. Real-time temperature during irradiation was recorded using an Infrared Thermal Imager (FLIR E76, American). In addition, the photothermal stability of Au_2Pt@4sMSN was further tested with a three-time ON/OFF cyclic irradiation experiment.

Cell Culture

The culture process Hepa 1-6 and NIH/3T3 cell lines, purchased from ATCC, was strictly followed according to the instructions. The cells were placed in a 37 °C and 5% CO2 cell culture incubator, and Dulbecco's Modified Eagle Medium (DMEM) medium containing 10 % FBS, 1 % PS, 1 % HEPES, and 1 % NFAA was changed once every two days. For the treatment of cellular experiments, the following groups were included to assess the effects of cellular behaviour: PBS, Au_2Pt, Au_2Pt@4sMSN, Au_2Pt@4sMSN-TPP, Au_2Pt@4sMSN/PS-TPP, Au_2Pt@4sMSN/PS-TPP + 808 nm laser (L1), Au_2Pt@4sMSN/PS-TPP + 660 nm laser (L2), and Au_2Pt@4sMSN/PS-TPP + L1 + L2.

Cytotoxicity

Hepa 1-6 and NIH/3T3 cells (5000 cells / well) were seeded onto a 96-well plate. After 24 h, the cells adhered to the plate, and different samples were added and continued to be incubated for 24 h. Then replace the medium with a 10 % CCK-8 solution. Following a further 3 h incubation, the optical density value at 450 nm was detected using a microplate reader.

Calcein-AM Staining

The Hepa 1-6 cells (a density of 300,000 cells per dish) were seeded onto the confocal dish and incubated with different treatments. Following the washing with PBS, the cells were further

incubated with fresh medium containing Calcein-AM and Propidium Iodide (PI) for 20 min. Then, the cells were imaged using the confocal microscope.

Cellular Uptake

A total of 20,000 cells were plated in confocal dishes. After 24 h, the cells adhered to the dishes, and the culture medium was then replaced with fresh medium containing either Au_2Pt@4sMSN/PS-TPP@CM or Au_2Pt@4sMSN/PS-TPP nanoparticles, followed by incubation for 3 and 6 hours. After incubation, the cells were washed with PBS, fixed, and imaged using confocal laser scanning microscopy (CLSM) to assess nanoparticle uptake.

Cell Flow Cytometry

The Hepa 1-6 cells (a density of 300,000 cells per dish) were cultured onto a 24-well plate. After 24 h, the cells adhered to the dishes, and the cells were co-incubated with Au_2Pt@4sMSN/PS-TPP@CM or Au_2Pt@4sMSN/PS-TPP for 2, 4, and 6 h. Then the cells were digested by trypsin and then collected for cytometry testing.

Motion study

The motility behavior of nanoparticles upon exposure to GSH was monitored and recorded using an inverted optical microscope. Time-lapse images were captured at defined intervals to track the movement of individual nanoparticles in real time. The acquired image sequences were then analyzed using ImageJ software equipped with the Manual Tracking plugin. Individual nanoparticle trajectories were manually tracked frame-by-frame to quantify their movement patterns. The resulting trajectory data were further processed to calculate mean square displacement (MSD) and diffusion coefficients.

Intracellular Glutathione Detection

The Hepa 1-6 cells (a density of 300,000 cells per dish) were cultured onto the confocal dish. After 24 h, the cells adhered to the dishes, changing the medium and then co-cultured with nanoparticles from different groups (Au_2Pt, Au_2Pt@4sMSN, Au_2Pt@4sMSN-TPP, and Au_2Pt@4sMSN-TPP) for 24 h. After that, 200 μL ThiolTracker™ Violet (Invitrogen) dye solution (10 μM) was replaced with the medium and further incubated for 30 min. Finally, Confocal microscopy was employed to image the cells and assess GSH depletion.

Intracellular Oxygen Detection

Hepa 1-6 cells (a density of 300,000 cells per dish) were seeded in a 24-well plate and incubated at 37°C for 24 hours. Following PBS washing, different nanoparticle formulations were introduced,

and the cells were continued to incubate for 24 hours. Afterward, $Ru(dpp)_3Cl_2$ was added, and the cells were incubated for an additional 30 min. Finally, fluorescence imaging was conducted to evaluate the intracellular oxygen levels.

Intracellular H_2S Detection

Hepa 1-6 cells (a density of 300,000 cells per dish) were cultured in confocal dishes. After 24 h, the cells adhered to the dishes, and different nano-formulations and WSP-5 fluorescence probe (50 nM) were co-cultured with cells for an additional 6 h. Finally, the cells were rinsed twice with PBS for confocal microscopy imaging.

Intracellular pH Detection

The fluorescent probe BCECF-AM was employed to assess intracellular pH variations. Briefly, following treatment with Au_2Pt, Au_2Pt@4sMSN, Au_2Pt@4sMSN-TPP, and Au_2Pt@4sMSN-TPP nano-formulations for 48 h, cells were incubated with 5 μM BCECF-AM at 37°C for 60 minutes to facilitate probe loading. Subsequently, cells were washed with PBS to remove excess dye, and CLSM was then utilized to visualize and analyse intracellular pH changes.

Mitochondria Targeting

Hepa 1-6 cells (a density of 300,000 cells per dish) were cultured in confocal dishes. After 24 h, the cells adhered to the dishes, Au_2Pt@4sMSN/PS-TPP and Au_2Pt@4sMSN/PS-TPP@CM at a concentration of 50 μg/mL were added and incubated for 3, 6, and 24 h. Mitochondria Tracker (1 μg/mL) was then added, and further co-incubated for 0.5 h. Subsequently, the cells were observed using confocal microscopy. For bio-TEM analysis, the cells were harvested by trypsinization and fixed in 2.5% glutaraldehyde at 4°C overnight. After standard dehydration, embedding, and ultrathin sectioning, the samples were observed with TEM.

JC-1 Staining

200,000 Hepa 1-6 cells were seeded into per confocal dish. After 24 hours of incubation, the medium was replaced, and the cells were co-cultured with nanoparticles from different groups (PBS, Au_2Pt, Au_2Pt@4sMSN, Au_2Pt@4sMSN-TPP, Au_2Pt@4sMSN-TPP/PS + L1, Au_2Pt@4sMSN-TPP/PS+ L2, and Au_2Pt@4sMSN-TPP/PS + L1 + L2) for an additional 24 hours. The cells were then irradiated with L1 for 5 minutes (0.33 W/cm²) or L2 for 5 minutes (0.2 W/cm²) and further incubated for 6 hours. After that, 1 mL of staining working solution of JC-1 was added to replace the medium and further co-incubated for 0.5 h. After that, the cells were rinsed with PBS three times for fluorescence imaging.

MPTP Detection

200,000 Hepa 1-6 cells were seeded into per confocal dish. The next day, the medium was replaced, and the cells were co-cultured with nanoparticles from different groups for an additional 24 hours. Following L1 or L2 irradiation treatment, 1 mL staining working solution (calcein and $CoCl_2$) was replaced by the medium and incubated for 30 min. Finally, the cells were stained with Hoechst 33342 and imaged using confocal microscopy.

ROS Detection

200,000 Hepa 1-6 cells were seeded into per confocal dish. After 24 hours of incubation, the cells adhered to the dishes, and the medium was replaced with various nanoparticles for co-culturing for 24 h. Following NIR 808 nm or NIR 660 nm laser irradiation, 1 mL fresh medium containing the working solution of DCFH-DA (10 μM) was added to and co-incubated for 30 min. Finally, the cells were stained with Hoechst 33342 and imaged using confocal microscopy.

CRT and HMGB1 Immunofluorescence Staining

The Hepa 1-6 cells were cultured onto the confocal dish at a density of 300,000 cells per dish. After different treatments, the cells were fixed with 4 % Paraformaldehyde (PFA). Then the cells were incubated with antibody CRT (1:200) and HMGB1 (1:200) overnight at 4 °C. Finally, the cells were incubated with DAPI solution for 5 min at room temperature and then observed with a confocal microscope.

ATP Determination

ATP levels of cells were measured with an Enhanced ATP Assay Kit (Byotime, Shanghai). Briefly, 200 μL of ATP lysis buffer was used to collect the cells that had been co-cultured with different nano-formulations. The samples were then centrifuged to collect the supernatant. The supernatant was mixed with the ATP detection reagent and measured using a chemiluminescence detector.

Western Blot

Following various treatments, Hepa 1-6 cells were lysed in RIPA buffer. Then, Protein concentrations were quantified, and 30 μg of total protein from various groups was separated on a precast SDS-PAGE gel (10%, Bio-Rad) under 120 V for 1 hour. After electrophoresis, the proteins were transferred onto PVDF membranes using a Bio-Rad Trans-Blot SD system with transfer buffer at 300 mA for 90 minutes. Then the PVDF membranes with proteins were subsequently blocked with 5% milk for 60 min at room temperature. Immunoblotting was performed by sequential incubation with primary antibodies HSP70 (1:1000), Cytochrome C (1:500), Cleaved-

Caspase-3 (1:500), β-actin (1:1000), and Cleaved-Caspase-9 (1:500) at 4°C overnight. Subsequently, the membrane was washed three times with TBS-T and then incubated with secondary antibody (1:2000) for 1 hour at room temperature. Finally, protein bands were visualized using the ChemiDoc Imaging System (Bio-Rad) following enhanced chemiluminescence detection.

Construction of Orthotopic Hepatocellular Carcinoma Model

An orthotopic liver cancer model was established using C57BL/6 (SPF, 4–5 weeks old, 15–20 g). Following the abdominal skin disinfection, the mice were anaesthetized with 3% sodium pentobarbital. Once the mice were fully anaesthetized, a laparotomy was performed to expose the liver. An insulin syringe was used to inject 100 μL of Hepa1-6 cells (Luciferase-labelled, 1×10^5 cells) into the liver tissue. The abdominal incision was closed with silk sutures and disinfected with povidone-iodine. Mice were then placed on a heating pad for recovery and fed under normal conditions (humidity 50–60%, temperature 22–26 °C) once fully awake. After 7 days, luciferin was injected into all mice to assess tumour size using In Vivo Imaging System (IVIS LUMINA III, American), and those with successful model establishment were selected for further experiments. All animal studies were approved by the Welfare and Ethics Committee of Lishui University Experimental Animal Centre (Approval No: 2025D306). Following the approved animal use protocol, the study adhered to strict humane endpoints, including self-harm, tumours exceeding 10% of body weight, and body weight loss greater than 20%. Euthanasia was conducted under anaesthesia through cervical dislocation if any of these conditions occurred. Additionally, the tumours were carefully monitored to ensure that the volume was within ethically acceptable limits.

***In vivo* Biodistribution and Infrared Thermography**

To evaluate the *in vivo* biodistribution of nano-formulations, two groups, Au_2Pt@4sMSN-TPP/Cy5.5 and Au_2Pt@4sMSN/Cy5.5-TPP@CM, were used for analysis of the targeting efficiency. Briefly, 100 μg/mL nano-formulations were injected via the tail vein, and the mice were vivo imaged in vivo at 1 h, 12 h, 24 h, and 48 h time points. The heart, liver, spleen, lung, and kidney were also collected and imaged to assess the targeting effect. To further evaluate *in vivo* photothermal conversion performance, the mice were treated with 100 μl PBS and Au_2Pt@4sMSN/PS-TPP@CM and then exposed to 1 W cm^{-2} 808 nm irradiation for 5 min. The temperature change was monitored with the FLIR E76 Infrared Thermal Imager.

In vivo Anticancer Treatment and Histopathology Staining

The mice with orthotopic Hepatocellular carcinoma were randomly divided into five groups, four mice in each group: (I) PBS; (II) Au_2Pt@4sMSN/PS-TPP@CM; (III) Au_2Pt@4sMSN/PS-TPP@CM + L1; (IV) Au_2Pt@4sMSN/PS-TPP@CM + L2; (V) Au_2Pt@4sMSN/PS-TPP@CM + L1 + L2. 100 μg/mL nano-formulations of different groups were injected into the tail vein on the first day. After 12 h, the mice of the laser treatment group were irradiated with the 808 nm laser (0.5 W cm^{-2}, 5 min) and the 660 nm laser (0.5 W cm^{-2}, 5 min). During the implementation of mild photothermal therapy, continuous temperature monitoring was conducted at the tumour site in mice using real-time infrared thermographic imaging, ensuring precise control to maintain the temperature at approximately 42 °C. The treatment was performed every 3 days, and the tumour size was recorded with a bioluminescence image every 4 days. The mice were sacrificed on day 15, and the hearts, livers with tumour, spleens, lungs, and kidneys were collected for subsequent histopathology analysis. Briefly, organ tissues fixed with paraformaldehyde were dehydrated, embedded in paraffin, and cut into thin slices (5 μm). The proteinase K was used to blot the tissue slices with an incubation at 37 °C for 30 min. Then the slides were stained with hematoxylin-eosin (HE), Ki67 (1:1000), TUNEL reaction mixture (Servicebio, China), HSP70 (1:1000), Cytochrome C (1:500), ROS probe (Bestbio, China), and Cleaved-Caspase 3 (1:500) at 37 °C for 60 min. After washing three times with PBS (pH 7.4) for 5 min each, the slides were incubated with the secondary antibody for 60 min. Finally, the slides were cover slipped using neutral balsam and imaged with an Inverted fluorescence microscope (Axio Observer, Zeiss).

Statistics and reproducibility

Origin 2024 b and GraphPad Prism 9.5 were used to conduct data analysis (unpaired t-test and one-way ANOVA), and the results are presented as means ± standard deviation (SD). The data were categorized based on p-values: (*) for $p < 0.05$, (**) for $p < 0.01$, (***) for $p < 0.001$, (****) for $P < 0.0001$. The analysis included all collected samples and animals, with no exclusions.

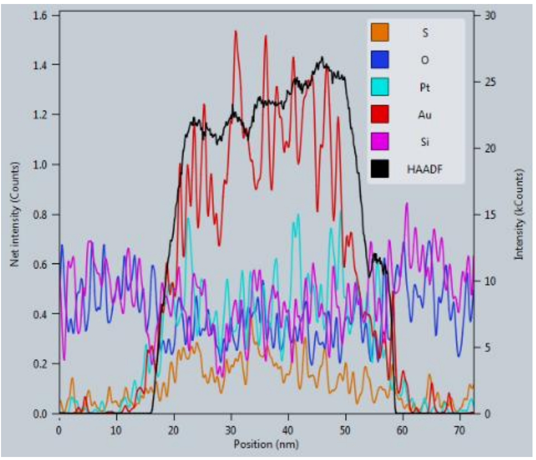

Figure S1. The element line-scanning profile of Au_2Pt@4sMSN.

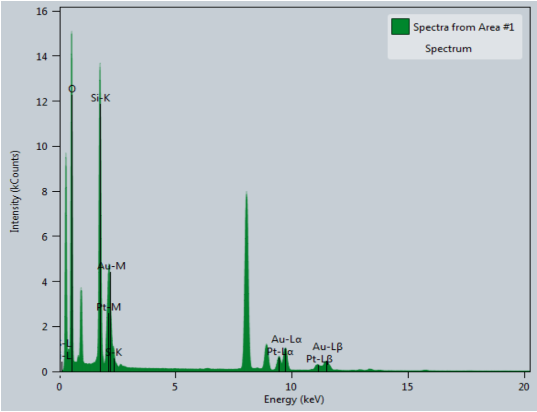

Figure S2. EDS spectrum of Au_2Pt@4sMSN.

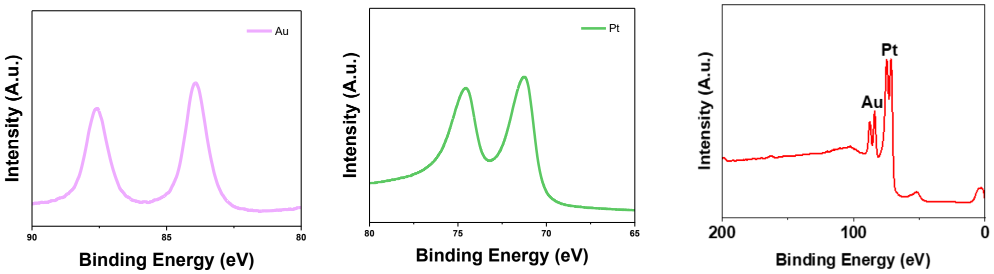

Figure S3. XPS spectra of Au_2Pt.

Title	Name	P At. %
**C1s Scan**	C1s	25.74
**O1s Scan**	O1s	11.35
**Pt4f Scan**	Pt4f	29.73
**Au4f Scan**	Au4f	11.96
**Si2p Scan**	Si2p	16.12
**S2p Scan**	S2p	5.11

Figure S4. Quantitative analysis of major elemental composition by XPS.

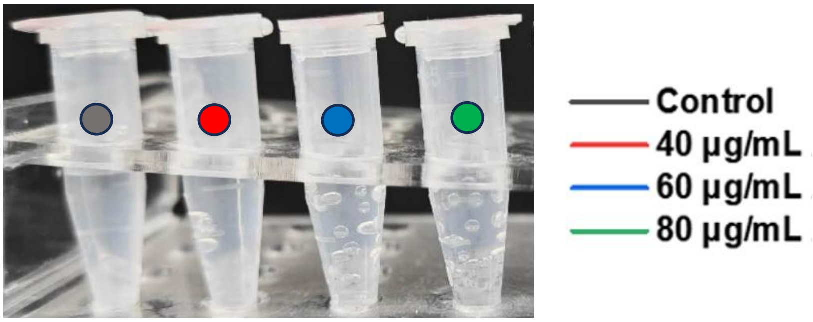

Figure S5. Photographic images of the generation of H_2S. The GSH concertation is 100 mM.

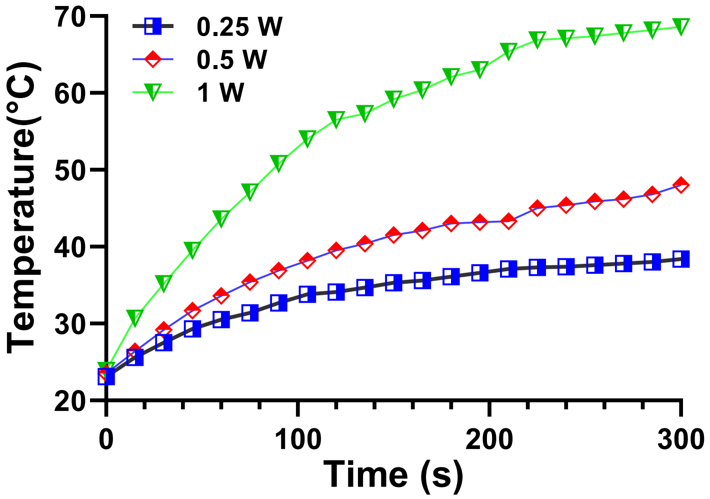

Figure S6. The temperature change curves of the 0.5 mg/mL solution under 808 nm laser irradiation at 0.25 W, 0.50 W, and 1 W.

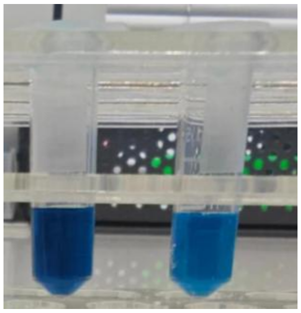

Figure S7. The comparison of POD activity between Au_2Pt and $Au_2Pt@WO$.

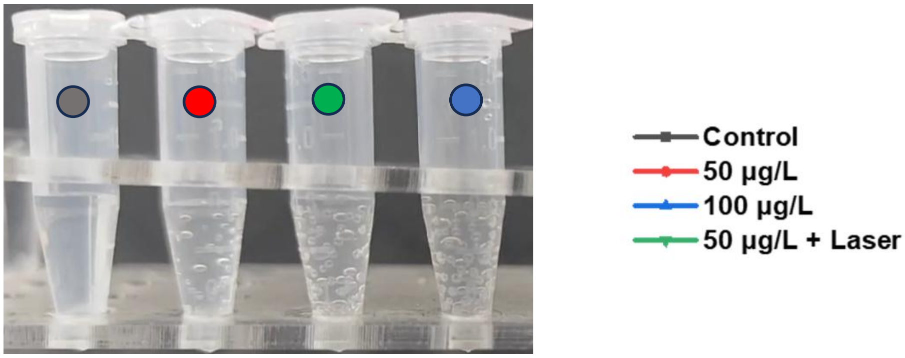

Figure S8. Photographic images of the generation of O_2.

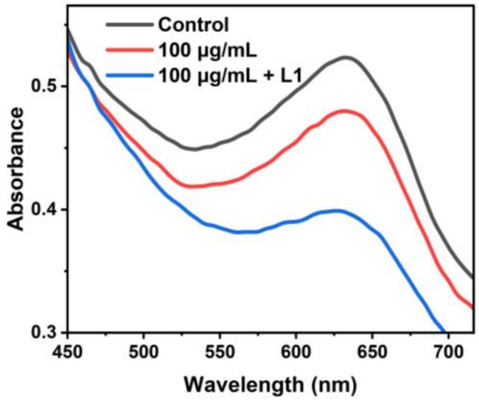

Figure S9. GOx-like enzyme activity of Au_2Pt@WO under 808 nm laser irradiation.

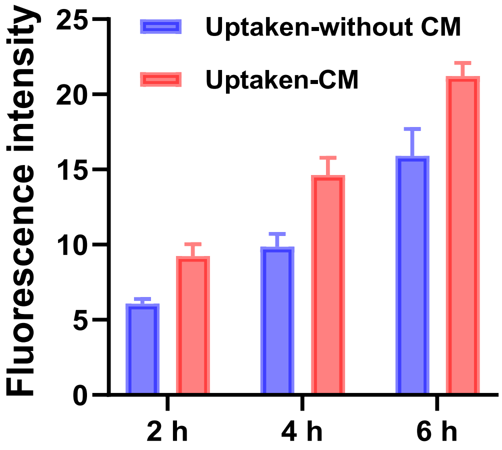

Figure S10. Quantitative fluorescence intensity analysis of cellular uptake images.

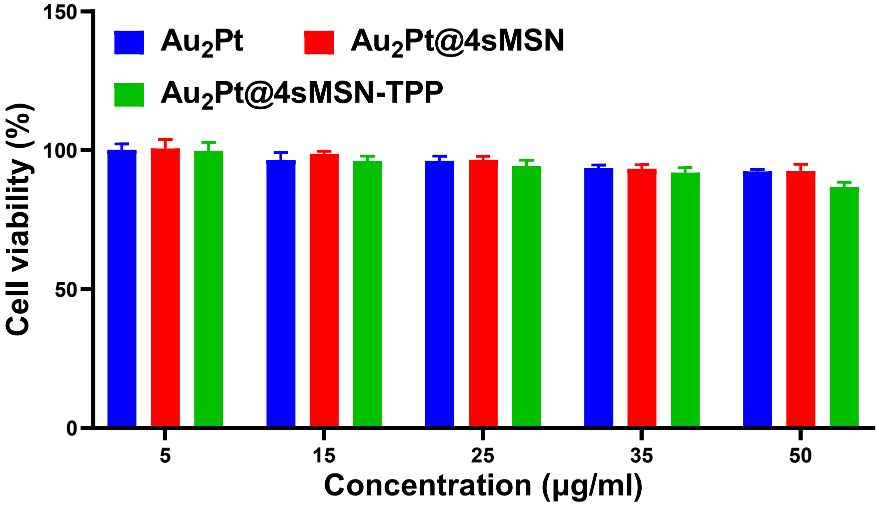

Figure S11. The cell viability of NIH/3T3 cells with different treatments.

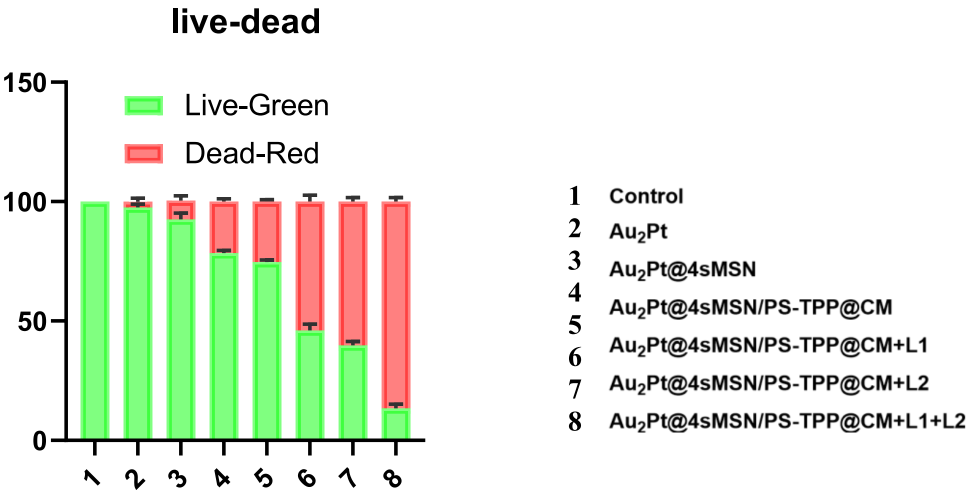

Figure S12. Fluorescence intensity quantification of live/dead stained cells.

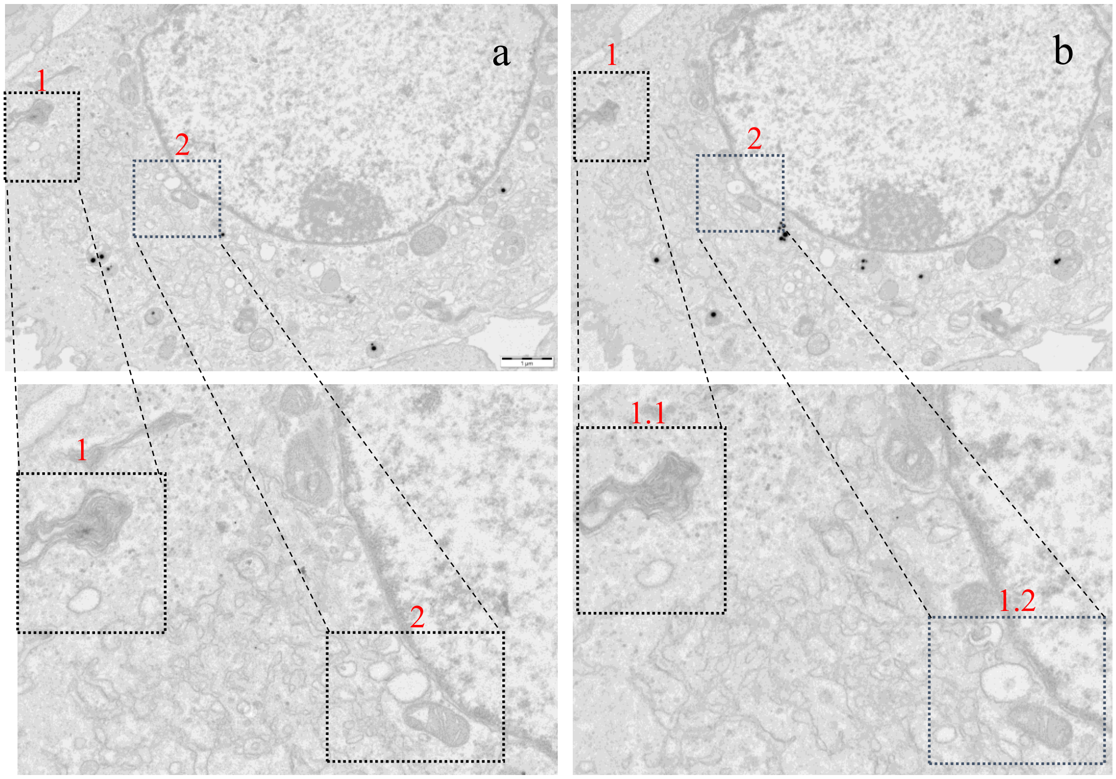

Figure S13. Bio-TEM images of serial ultrathin sections (70 nm) from the targeted group. The black dashed rectangles indicate sequential sections of the same cell.

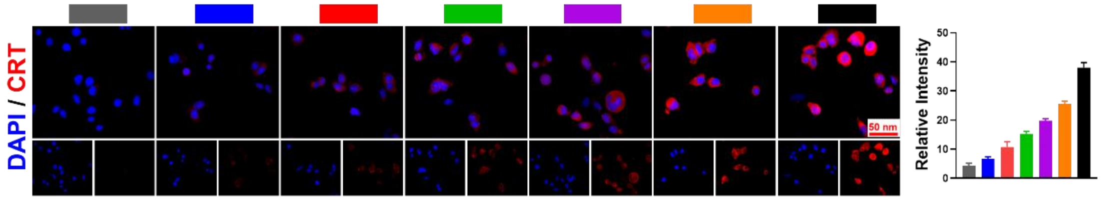

Figure S14. Fluorescence images and analysis of CRT expression.

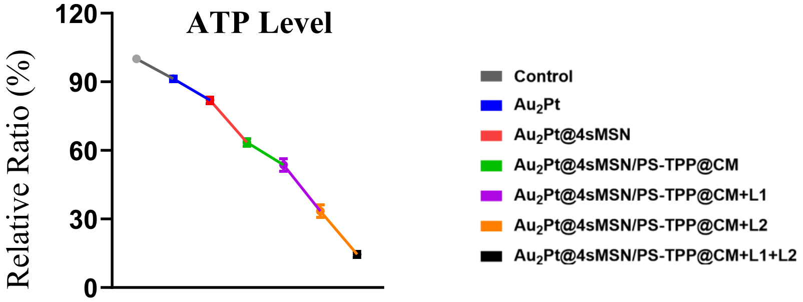

Figure S15. ATP quantitative analysis of different groups.

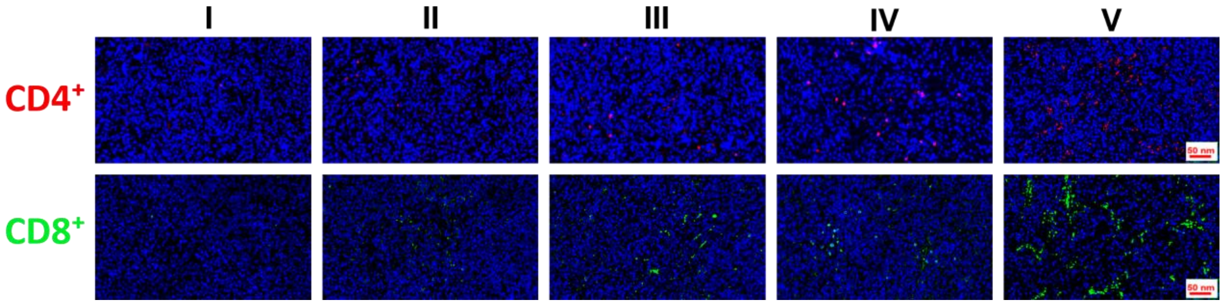

Figure S16. Representative immunofluorescence images of tumor-infiltrating $CD4^+$ (red) and $CD8^+$ (green) T cells.

[1] M. Wang, M. Chang, Q. Chen, D. Wang, C. Li, Z. Hou, J. Lin, D. Jin, B. Xing, *Biomaterials* **2020**, 252, 120093.

[2] X. Ma, W. Zhou, R. Zhang, C. Zhang, J. Yan, J. Feng, J. M. Rosenholm, T. Shi, X. Shen, H. Zhang, *Mater. Today Bio* **2023**, 20, 100663.

[3] K. M. Dubowski, *Clin. Chem.* **2008**, 54, 1919.

[4] C. Zhang, H. Wang, X. Yang, Z. Fu, X. Ji, Y. Shi, J. Zhong, W. Hu, Y. Ye, Z. Wang, D. Ni, *Sci. Adv.* **2022**, 8, eabp9882.
